# Stimulation of mTORC1 with L-leucine Rescues Defects Associated with Roberts Syndrome

**DOI:** 10.1371/journal.pgen.1003857

**Published:** 2013-10-03

**Authors:** Baoshan Xu, Kenneth K. Lee, Lily Zhang, Jennifer L. Gerton

**Affiliations:** 1 Stowers Institute for Medical Research, University of Kansas School of Medicine, Kansas City, Kansas, United States of America; 2 Department of Biochemistry and Molecular Biology, University of Kansas School of Medicine, Kansas City, Kansas, United States of America; University of Pennsylvania, United States of America

## Abstract

Roberts syndrome (RBS) is a human disease characterized by defects in limb and craniofacial development and growth and mental retardation. RBS is caused by mutations in ESCO2, a gene which encodes an acetyltransferase for the cohesin complex. While the essential role of the cohesin complex in chromosome segregation has been well characterized, it plays additional roles in DNA damage repair, chromosome condensation, and gene expression. The developmental phenotypes of Roberts syndrome and other cohesinopathies suggest that gene expression is impaired during embryogenesis. It was previously reported that ribosomal RNA production and protein translation were impaired in immortalized RBS cells. It was speculated that cohesin binding at the rDNA was important for nucleolar form and function. We have explored the hypothesis that reduced ribosome function contributes to RBS in zebrafish models and human cells. Two key pathways that sense cellular stress are the p53 and mTOR pathways. We report that mTOR signaling is inhibited in human RBS cells based on the reduced phosphorylation of the downstream effectors S6K1, S6 and 4EBP1, and this correlates with p53 activation. Nucleoli, the sites of ribosome production, are highly fragmented in RBS cells. We tested the effect of inhibiting p53 or stimulating mTOR in RBS cells. The rescue provided by mTOR activation was more significant, with activation rescuing both cell division and cell death. To study this cohesinopathy in a whole animal model we used ESCO2-mutant and morphant zebrafish embryos, which have developmental defects mimicking RBS. Consistent with RBS patient cells, the ESCO2 mutant embryos show p53 activation and inhibition of the TOR pathway. Stimulation of the TOR pathway with L-leucine rescued many developmental defects of ESCO2-mutant embryos. Our data support the idea that RBS can be attributed in part to defects in ribosome biogenesis, and stimulation of the TOR pathway has therapeutic potential.

## Author Summary

Roberts syndrome is a human developmental disorder caused by mutations in the ESCO2 gene. This gene encodes an acetyltransferase that acetylates the cohesin ring complex to promote a locked configuration. The cohesin complex binds to many locations on chromosomes and mutations that affect its function result in changes in gene expression. In fact, Roberts syndrome and other diseases caused by mutations in cohesin are associated with differential gene expression. We wanted to understand how mutations in ESCO2 affect two important molecular pathways that detect cellular stress, the p53 and mTOR (mammalian target of rapamycin) pathways. We report that mutations in ESCO2 are associated with p53 activation and inhibition of mTOR in human cells and zebrafish. We tested the rescue effect of p53 inhibition and mTOR activation on human Roberts syndrome cells and zebrafish models for Roberts syndrome. While both treatments displayed rescue effects, the activation of mTOR provided more significant rescue. Our work suggests that stimulation of the mTOR pathway with the amino acid L-leucine has therapeutic potential for Roberts syndrome. In addition, our work suggests that some of the differential gene expression in Roberts syndrome may be explained by translational inhibition connected with the inhibition of the mTOR pathway.

## Introduction

Cohesin is a protein complex that adheres sister chromatids from the time of their replication until their division [1], [2]. Cohesion between sister chromatids is facilitated by acetylation of the Smc3 subunit of the complex by the *ECO1* acetyltransferase during S phase [3], [4], [5]. Human developmental syndromes such as Roberts syndrome (RBS) and Cornelia de Lange syndrome, termed cohesinopathies, arise from mutations in cohesin genes [6]. ESCO2, which is a human ortholog of *ECO1* in the yeast *Saccharomyces cerevisiae*, is inactivated in RBS [7].

RBS is an autosomal recessive, multi-system disorder characterized by prenatal growth retardation (ranging from mild to severe), limb malformations (including bilateral symmetric tetraphocomelia or hypomelia caused by mesomelic shortening), craniofacial abnormalities and mental retardation [8], [9], [10], [11], [12]. Previous studies have reported loss of ESCO2 acetyltransferase activity in RBS [13]. Chromosomes show a characteristic pattern of heterochromatin repulsion with the regions affected including centromeres and NORs (nucleolar organizing centers or rDNA). A previous report revealed that mutations in yeast *ECO1* and human ESCO2 impaired ribosomal RNA (rRNA) production and protein synthesis in budding yeast and human immortalized RBS cells [14]. Also, mutations in cohesin are associated with aberrant nucleolar morphology in yeast [15]. Cohesin binds to the rDNA in every organism studied, giving cohesin the potential to affect the structure and function of the nucleolus. We hypothesized that defective ribosome biogenesis contributes to the etiology of the RBS disorder.

Perturbation of ribosome biogenesis is thought to lead to nucleolar stress and p53 activation. The mechanism appears to be the specific binding of ribosome proteins to Mdm2, which inhibits its E3 ubiquitin ligase function toward p53, leading to p53 stabilization and activation [16], [17], [18]. This binding happens when there is an imbalance of ribosomal proteins. Once p53 is stabilized, it will act to promote the transcription of Mdm2 in a feedback loop, as well as several other genes such as p21 and p27, cyclin-dependent kinase inhibitors [19]. Depletion of ribosomal proteins such as Rpl5, Rpl11, or Rps7 induces p53 upregulation in various cell lines [17], [20], [21], [22]. Loss of Rpl11 impaired zebrafish embryonic development via a p53-dependent apoptotic response [22]. Furthermore, defects in ribosomal proteins such as Rps6 (S6), Rps19 and Rpl24 have been implicated in congenital malformations and aberrant growth during fetal development [17], [18], [22], [23], [24], [25]. Taken together, these studies indicate a strong link between p53 activation and the process of ribosome biogenesis.

The TOR (target of rapamycin) pathway is a major node of control for protein translation and ribosome biogenesis. mTOR allows eukaryotic cells to adjust their protein biosynthetic capacity [26], [27], [28] through downstream effectors: (1) S6K1, a kinase that phosphorylates Rps6 and promotes protein synthesis and cell proliferation, and (2) eukaryotic translation initiation factor 4E-binding protein 1 (4EBP1), a protein that prevents translation when its unphosphorylated form interacts with eIF4E [29], [30], [31]. The TOR pathway can regulate intracellular processes such as transcription by RNA polymerase I [32], [33], [34] based on extracellular signals such as amino acid availability [35], [36], [37] or intracellular stress. mTORC1 can be stimulated by L-leucine through a mechanism that involves the leucyl tRNA synthase promoting the activity of GTP activating proteins that act on mTORC1 [38], [39]. Given the hypothesis that RBS is associated with defects in rRNA production and ribosome biogenesis [40], we wanted to test whether the TOR pathway was inhibited in RBS and if so, whether stimulation of TOR by L-leucine might rescue some of the defects associated with RBS.

In this study, we found that translational efficiency and rRNA production are impaired in primary human RBS cells. Furthermore, nucleoli are highly fragmented. RBS cells showed an activation of p53, and inhibition of mTOR. Inhibition of p53 activity with pifithrin-alpha (Pifα), or stimulation of mTOR with L-leucine (L-Leu) both partially rescued proliferation in RBS cells. L-Leu also partially improved rRNA production and protein synthesis of RBS cells. Using zebrafish models for RBS, we found similar p53 activation and TOR pathway inhibition. L-Leu partially stimulated the TOR pathway in zebrafish RBS models and partially rescued several aspects of development.

## Results

### Primary human RBS cells show poor proliferation, rRNA production, and protein synthesis

In this study, we used RBS cells from 3 different sources. Immortalized skin fibroblasts came from a two-month old male Roberts syndrome patient homozygous for the mutation 877_878 delAG in exon 4 (reported in [7]). De Winter and colleagues constructed a “corrected” RBS line in which the SV40 immortalized ESCO2-deficient fibroblasts were stably transfected with a cDNA construct encoding V5-tagged wild-type ESCO2 protein. Cytogenetic analysis of chromosomes demonstrated that the tagged ESCO2 protein compensated for the loss of ESCO2 activity in the mutant cells [41]. For untransformed primary fibroblasts, the donor subject was homozygous for a 5 bp deletion at nucleotide 307 in exon 3 of the ESCO2 gene (c.307_311delAGAAA) resulting in a frameshift that leads to a truncated protein (p.I102fsX1). For untransformed amniocytes, the donor subject was a compound heterozygote: one allele has a 1 bp deletion at nucleotide 752 in exon 3 of the ESCO2 gene (c.752delA) resulting in a frameshift leading to a premature stop codon and a predicted protein truncation (p.K253fsX12); the second allele has an A>G substitution in intron 6 [c.IVS6-7A>G (c.1132-7A>G)] which activates a cryptic splice site (p.I377_378insLX).

**Figure 1:**
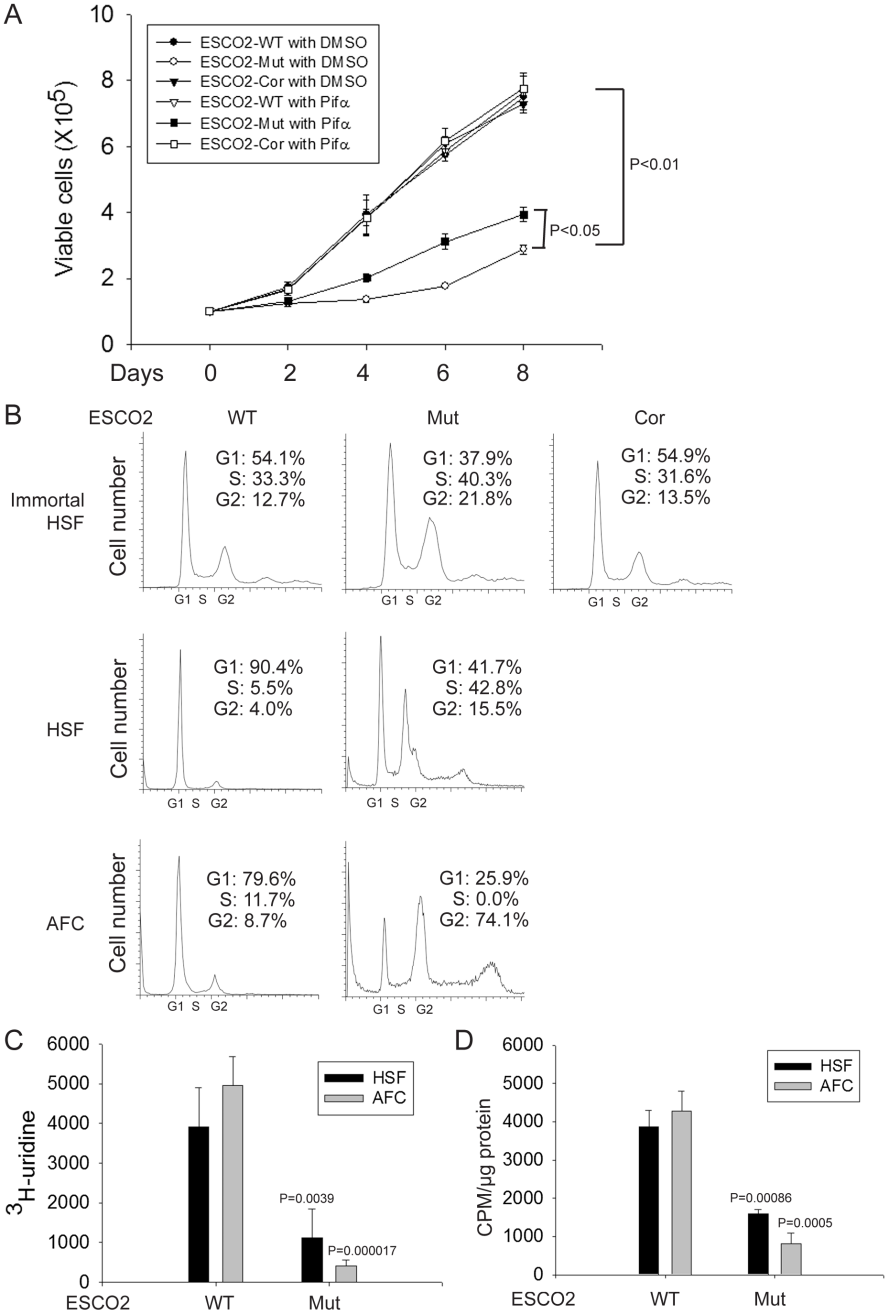
Human RBS cells showed severely slow proliferation and decreased protein translation. (A). Immortalized WT, RBS, and corrected cells were seeded into 6-well plates with 0.5×10^5^ cells/mL. After 8 days in culture, immortalized RBS cells showed very poor proliferation, compared with wild type (WT) and ESCO2-corrected (Cor) cells (P<0.01). Inhibition of p53, by Pifα (10 µM) incubation, partially rescued proliferation of RBS cells (P<0.05, 2-way ANOVA). (B). By FACScan analysis, RBS cells accumulated in G2/M phase. (C). Untransformed RBS and normal human skin fibroblasts (HSF) and amniotic fluid-derived cells (AFC) were grown in DMEM plus 15% FBS. ^3^H-uridine (5 µCi) was incubated with 10^6^ cells from each group for two hours. Total RNA was isolated with TriZol reagent (Invitrogen, U.S.A) and the concentration of each RNA sample was measured by OD_260/280_. 1 µg of each sample was counted in a Beckman LS 6500 multipurpose scintillation counter to determine the amount of ^3^H-uridine incorporated. Four independent cultures were labeled to derive the standard deviation. Significance relative to normal cells was calculated using an unpaired t test. P = 0.0039, HSF ESCO2-Mut *vs* HSF ESCO2-WT; P = 0.000017, AFC ESCO2-Mut *vs* AFC ESCO2-WT. (D). Equal numbers of untransformed RBS and normal HSF and AFC cells were grown in DMEM plus 15% FBS. Cells were washed in PBS twice, switched to 3 mL Met/Cys-free Dulbecco's modied Eagle's medium containing 10 µM MG-132, a proteasome inhibitor, and pulsed with 30 µCi of ^35^S-methionine for 4 hrs. Cells were lysed in RIPA buffer and proteins were precipitated by the addition of hot 10% TCA. After centrifugation, the precipitate was washed twice in acetone. The precipitate was dissolved in 100 µL of 1% SDS and heated at 95°C for 10 min. An aliquot of the SDS extract was counted in Esoscint for ^35^S radioactivity in a liquid scintillation spectrometer to determine the amount of ^35^S-methionine incorporated into proteins. P = 0.00086, HSF ESCO2-Mut *vs* HSF ESCO2-WT; P = 0.0005, AFC ESCO2-Mut *vs* AFC ESCO2-WT.

Production of ribosomal RNA (rRNA) and protein synthesis are reduced and there are fewer actively translating ribosomes in *eco1* mutant budding yeast and human immortalized RBS fibroblasts [14]. To extend this work, we conducted similar experiments with primary human RBS cells and carefully monitored their proliferation. Proliferation of three different sources of RBS cells (immortalized fibroblasts, primary skin cells, and primary amniotic fluid cells, described above) was much slower than wild type (WT) cells (data shown only for immortalized cells, Figure 1A). Morphologically, the RBS AFCs appeared longer than normal AFCs (data not shown). Re-introduction of ESCO2 to the immortalized line, indicated as ESCO2-corrected RBS cells, rescued proliferation. To further examine proliferation, we used FACScan to detect the cell cycle profile for these cell lines. There was an increase in G2/M cells in the RBS lines (greater than 2 fold increase for immortalized fibroblasts and over 10 fold increase for untransformed AFC cells) (Figure 1B). Additionally, we performed ^3^H-uridine labeling to measure total rRNA production and ^35^S-methionine incorporation to quantify protein synthesis in the untransformed RBS cells. The results showed both rRNA and protein synthesis were significantly downregulated (Figure 1C–D), similar to the previous report for the immortalized RBS cells. These data collected from three independent sources of RBS cells suggest that reduced (1) proliferation with a G2/M delay, (2) rRNA production, and (3) protein synthesis are general features of RBS cells.

### p53 activation and nucleolar fragmentation are features of human RBS cells

**Figure 2:**
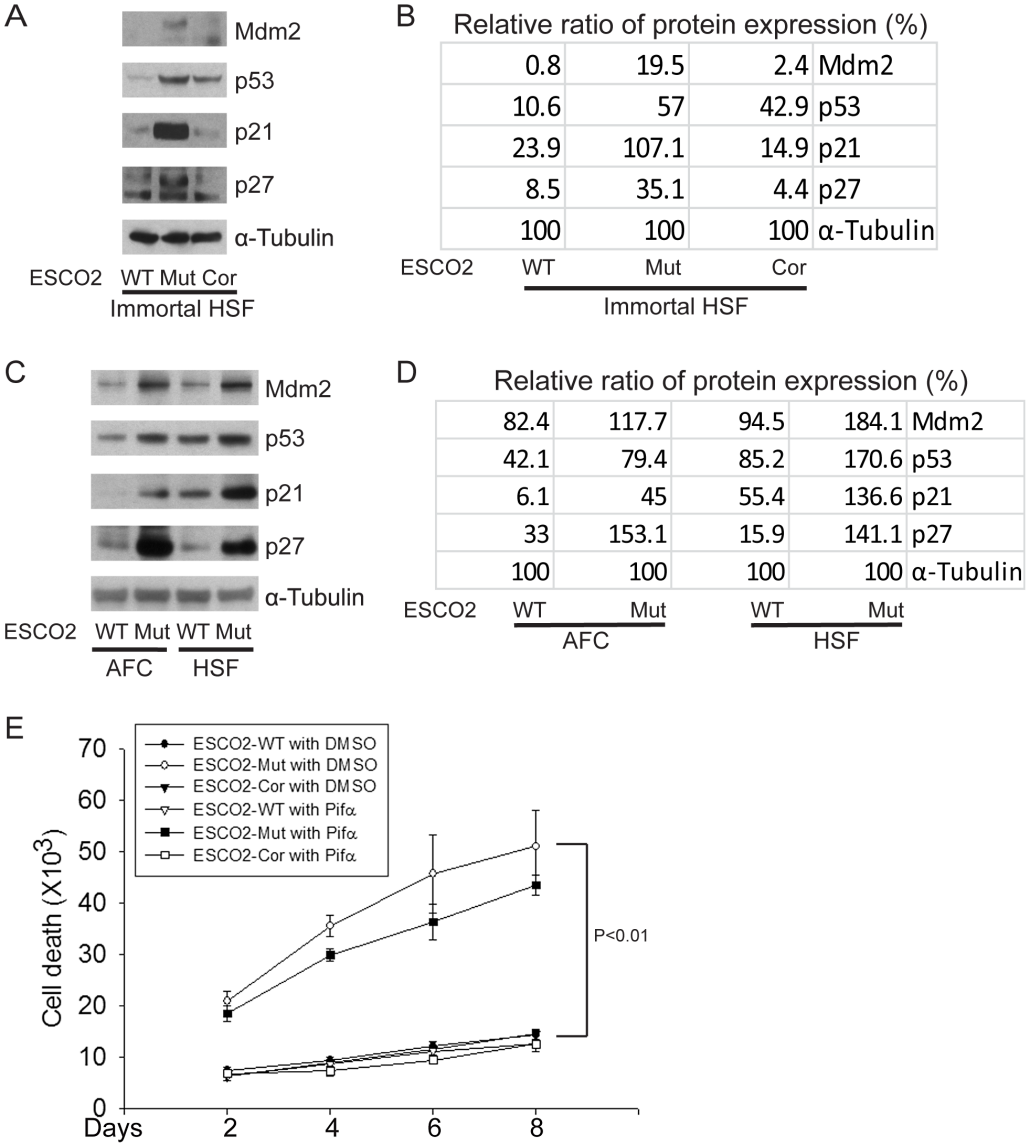
The p53 pathway is upregulated in RBS cells. (A–D). Western blot analysis showing p53, Mdm2, p21 and p27 were markedly activated in both immortalized (A–B) and untransformed RBS cells (C–D). Re-introduction of ESCO2 only partly reduced p53 activation in the immortalized RBS cells (A–B). The relative ratio of protein expression was quantified with ImageQuant TL software in (B, D). (E). Cell death was quantified by counting the number of trypan blue positive cells. Pifα incubation did not rescue cell death in human immortalized RBS cells.

The cell cycle delay and the previously reported sensitivity to DNA-damaging agents in human RBS cells [41] prompted the question whether p53 activation might contribute to poor proliferation in RBS cells. p53 activation has been previously reported for zebrafish models of RBS [42]. By Western blot analysis, we found p53 was activated in immortal and untransformed RBS cells, and the levels of proteins encoded by genes positively regulated by p53 are higher in the mutant cells as well, as represented by induction of the targets Mdm2, p21, and p27 (Figure 2A–D).

To test whether p53 activation in RBS might be contributing to poor proliferation, WT and RBS cells were incubated with the p53 inhibitor pifithrin-alpha (Pifα, 10 µM). This concentration was selected based on reports in the literature [43], [44] as well as our own titrations (data not shown). Cell proliferation and survival were quantified every two days using a bright-line hemocytometer with trypan blue staining to discriminate viable and dead cells. At 8 days, cell counting indicated the number of RBS cells was about 36% compared to WT cells (normalized to 100%) (Figure 1A). This number will reflect both cell division and cell death. Inhibition of p53 activity partially restored proliferation of RBS cells as shown in the cell growth curve (Figure 1A), suggesting that upregulation of p53 contributes to proliferation defects. But Pifα treatment did not suppress the elevated rate of death of the RBS cells (Figure 2E). Overall, cell division and cell death both contributed to poor proliferation for RBS cells (Figure 1A and Figure 2E), with cell death contributing approximately ∼15%. Pifα partially rescued the G2/M delay in the RBS cells, as shown in Figure S1A. However, Pifα did not rescue rRNA production or protein synthesis (Figure S1B–C).

**Figure 3:**
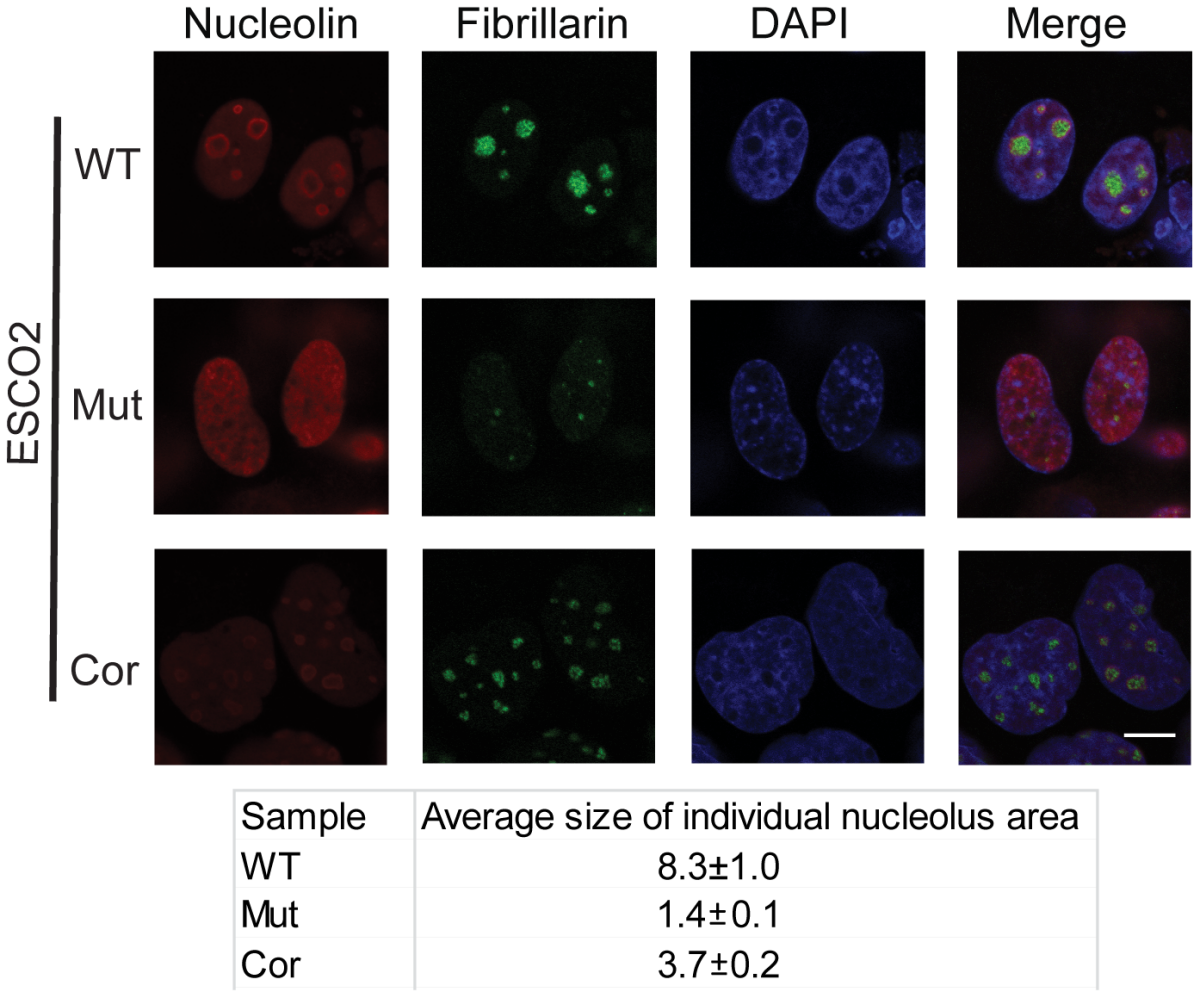
Nucleolar organization was severely disrupted in the immortalized RBS cells. WT, RBS, and corrected RBS cells were immunostained with antibodies to the nucleolar components nucleolin and fibrillarin, and imaged with confocal microscopy. Scale bar = 10 µm. DNA was stained with DAPI. The average size (µm^2^) of individual nucleoli was measured in the fibrillarin stained sample using Image J software. About 20 cells were quantified for each sample.

One mechanism by which p53 can become activated is nucleolar stress [17], [18], [20], [45]. Studies from mouse mutants showed that nucleolar disruption resulted in increased p53 levels and inhibition of mTOR activity, leading to mitochondrial dysfunction and increased oxidative stress, and contributing to neurodegenerative disease [46], [47], [48]. To examine the state of the nucleoli in RBS cells, we analyzed the distribution of the nucleolar proteins fibrillarin and nucleolin using immunofluorescence in the immortalized WT, ESCO2 mutant and corrected cells. These experiments showed that compared with WT cells and ESCO2 corrected cells, ESCO2 mutant cells have very fragmented signals for fibrillarin and very little distinct signal for nucleolin (Figure 3). Average size measurement of individual nucleolus area based on the fibrillarin staining showed that ESCO2 mutant cells have much smaller nucleolar size (1.4±0.1 µm^2^) compared with WT cells (8.3±1.0 µm^2^), but re-introduction of ESCO2 protein partially rescued the nucleolar size in the ESCO2 corrected cells (3.7±0.2 µm^2^). The highly fragmented nucleoli in RBS cells are consistent with the idea that p53 activation is caused in part by nucleolar stress. Furthermore, this result is consistent with our working model that cohesion at the rDNA may normally contribute to efficient nucleolar morphology and function. Inhibition of p53 did not rescue the aberrant nucleolar morphology in human RBS cells (Figure S2), suggesting that p53 activation may be a downstream consequence of the nucleolar fragmentation caused by ESCO2 mutation.

### mTOR signaling is impaired in RBS cells and can be stimulated with L-leucine

Given the defect in protein synthesis in RBS cells, we examined the state of the TOR (target of rapamycin) pathway. Experiments carried out with an *eco1-W216G* yeast mutant revealed that the growth of this mutant was more sensitive to rapamycin treatment than a WT strain (Figure S3A–B). This mutation has been associated with RBS and compromises the acetyltransferase activity of the protein [13], [49]. Since rapamycin inhibits the TOR pathway, this result suggested that the TOR pathway might already be partly compromised in the mutant background. The mRNA for *TOR1* was reduced 30% in the *eco1-W216G* mutant cells [14]. We asked whether the TOR pathway was affected in human RBS cells.

**Figure 4:**
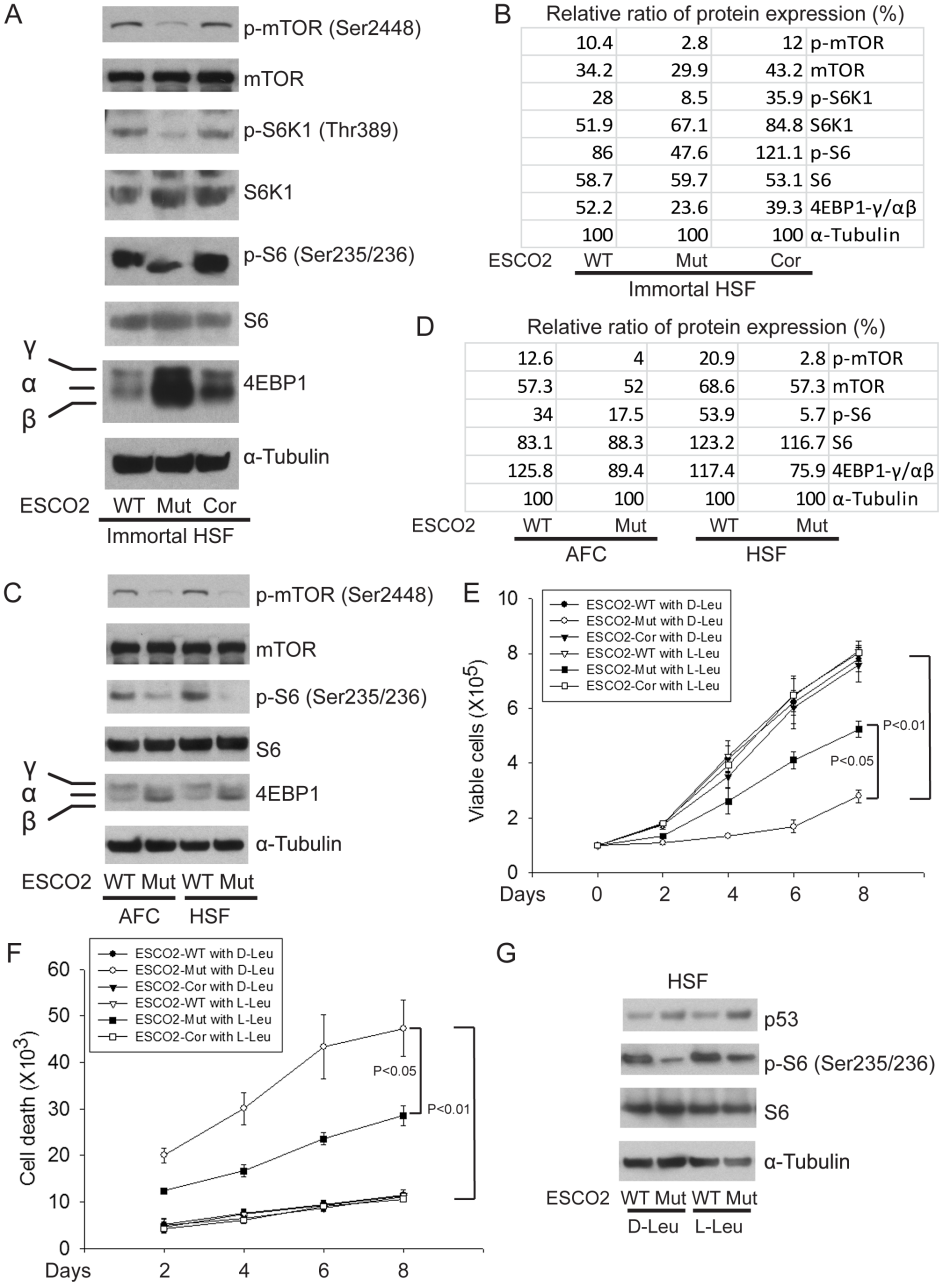
ESCO2 mutation is associated with mTOR inhibition in RBS cells. (A–D). Phosphorylation of S6K1/S6 and 4EBP1-γ subunit ratio divided by α/β subunit was downregulated in the human immortalized RBS cells (A–B) and primary cells (C–D), as measured by Western blot analysis. The results are representative of three independent experiments. Total levels of S6, S6K1, and tubulin serve as loading controls. The relative ratios of protein expression were quantitated with ImageQuant TL software in (B, D). (E). L-Leu application partially improved poor proliferation of immortalized RBS cells. Each plot represents the average ± SEM of the ratio of the measurement of the indicated cell number, as calculated for three independent samples for 2-way ANOVA statistical analysis. P<0.01, HSF ESCO2-Mut with 10 mM D-Leu *vs* HSF ESCO2-WT with10 mM D-Leu; P<0.05, HSF ESCO2-Mut with 10 mM D-Leu *vs* HSF ESCO2-Mut with 10 mM L-Leu. (F). Elevated levels of cell death were partially suppressed by L-Leu supplementation. P<0.01, HSF ESCO2-Mut with 10 mM D-Leu *vs* HSF ESCO2-WT with 10 mM D-Leu; P<0.05, HSF ESCO2-Mut with 10 mM L-Leu *vs* HSF ESCO2-Mut with 10 mM D-Leu. (G). L-Leu, but not D-Leu, partially rescued the phosphorylated form of S6 in primary RBS fibroblasts. p53 levels were not rescued by treatment with L-Leu.

Whole cell extracts were made from immortalized WT, ESCO2-mutant, ESCO2-corrected human fibroblasts, and the two untransformed human normal and RBS cell lines, all from Figure 1. The concentrations of total protein were measured to normalize all samples and tubulin was used as a loading control. Extracts were used for Western blotting to measure individual members of the mTOR signaling pathway associated with protein translation. As shown in Figure 4A–D, ribosomal S6 kinase (Thr389 of S6K1, a site regulated by mTOR [50], [51]) was hypophosphorylated in the ESCO2 mutant strains, which is associated with reduced activity. Consistently, phosphorylation of ribosomal protein S6 (S6) at Ser235/236, which is a direct target of S6K1 and a component of the 40S ribosomal subunit, was reduced in ESCO2 mutant strains. This phosphorylation event normally promotes protein translation. Notably, phosphorylation of mTOR at Ser2448, which is regulated by amino acid availability/energy status [52], Akt [53], [54] and cellular damage stress [55] was low, indicating that mTOR activity is impaired in RBS cells.

The effect of ESCO2 mutation on the phosphorylation state of Eukaryotic translation initiation factor 4E-binding protein 1(4EBP1) was examined. 4EBP1 is one member of a family of translational repressor proteins. The protein directly interacts with eukaryotic translation initiation factor 4E (eIF4E), which is a limiting component of the multi-subunit complex that recruits 40S ribosomal subunits to the 5′ end of mRNAs. Interaction of this protein with eIF4E inhibits complex inhibits assembly and represses translation. Phosphorylation results in its dissociation from eIF4E and activation of mRNA translation. Phosphorylation of 4EBP1 decreases its electrophoretic mobility during SDS-polyacrylamide gel electrophoresis. In RBS cells, the faster mobility band α/β was dramatically increased that corresponds to the unphosphorylated form of 4EBP1. This indicates that 4EBP1 exists in a state that will inhibit translation. Previous polysome analysis has shown that the fraction of actively translating ribosomes is reduced in both an *eco1-W216G* mutant yeast strain and RBS cells [14]. Therefore, the data collectively support the idea that translation and the TOR pathway are inhibited in RBS cells.

We wondered whether stimulation of protein synthesis could rescue proliferation of RBS cells. L-leucine (L-Leu) has been reported to enhance translation initiation and protein synthesis [56] via induction of the mTOR pathway [57], [58], [59]. We supplemented the culture medium with 10 mM L-Leu or D-leucine (D-Leu) up to 8 days, and cell proliferation was quantified using a bright-line hemocytometer with trypan blue staining to discriminate viable and dead cells. The culture medium also contained other amino acids, such as L-glutamine (L-Glu), which helps with the uptake of L-Leu. L-Leu significantly rescued proliferation of RBS cells (Figure 4E). Both cell death (Figure 4F) and the G2/M delay (Figure S4A) were partially suppressed. Immunoblot analysis confirmed that phosphorylation of S6 in RBS cells was partially rescued by L-Leu treatment, but p53 was not altered (Figure 4G). Notably, L-Leu supplementation in WT cells did not increase the levels of phosphorylated S6 (Figure 4G), which could indicate that the TOR pathway is already fully active. Moreover, ^3^H-uridine and ^35^S-methionine metabolic labeling experiments indicated that the defects in rRNA production and protein synthesis in RBS cells were also partially restored by L-Leu (Figure S4B–E). Rapamycin treatment curtailed the effect (Figure S4C, E), suggesting L-Leu is acting through mTOR. Thus, the proliferation defects associated with RBS could be partially corrected through L-Leu stimulation of the mTOR pathway and translational induction. However, L-Leu treatment did not rescue nucleolar fragmentation (Figure S5). We speculate that the fragmentation is a molecular defect caused by ESCO2 mutation; L-Leu may stimulate mTOR and its downstream effectors without rescuing nucleolar fragmentation or p53 activation.

We wanted to explore upstream effectors of mTOR in RBS cells. AMP-activated kinase (AMPK) activity can impede mTORC1 signaling through tuberous sclerosis complex (TSC) 1/2 as a repressor of mTOR function [55], [60]. TSC2 has GTPase-activating protein (GAP) activity toward the Ras family small GTPase Rheb (Ras homolog enriched in brain), and TSC1/2 antagonizes the mTOR signaling pathway via stimulation of GTP hydrolysis of Rheb [61], [62], [63], [64], [65], [66]. AMPK activates TSC2 phosphorylation to catalyze the conversion of Rheb-GTP to Rheb-GDP and thus inhibits mTOR [67]. In two different sources of RBS cells, AMPK, its substrate Acetyl-CoA carboxylase (ACC), and TSC2 (Ser1387) were phosphorylated (Figure S6A–B). Thus, TOR inhibition may be due in part to these upstream effectors.

In some cancer cell lines, p53 activation triggers downregulation of the mTOR pathway [68], [69], but p53 and mTOR are not connected in all types of cells [55], [70]. The phosphorylation of AMPK, ACC, and TSC2 were only slightly attenuated with siRNA directed at p53 (Figure S6C), suggesting that the signals that are promoting the inhibition of mTOR are at least partly independent of p53 activation. This is consistent with the finding that L-Leu stimulation of mTOR did not reduce p53 activation, but boosted proliferation, rRNA production, and protein synthesis. mTOR can physically interact with RNA polymerase I and III promoters and stimulate transcription [71], which could partly compensate for the poor production of rRNA and ribosomes associated with ESCO2 mutation.

The phosphorylation of AMPK can be triggered by an increased AMP to ATP ratio [72] which can be caused by cellular stress. We found evidence for an increase in reactive oxygen species (ROS) associated with RBS cells (Figure S6D–E). The ROS could be produced as part of the stress associated with mutations in ESCO2. For example, ESCO2 mutant cells have defects in DNA replication and DNA damage repair [41], [73], and in this report we demonstrate defects in nucleolus formation. Any or all of these could contribute to the production of ROS [46], [74], [75], [76]. Our results suggest that ESCO2 mutation is associated with oxidative stress, and this could contribute to the inhibition of mTOR.

### Zebrafish RBS models show inhibition of the TOR pathway

**Figure 5:**
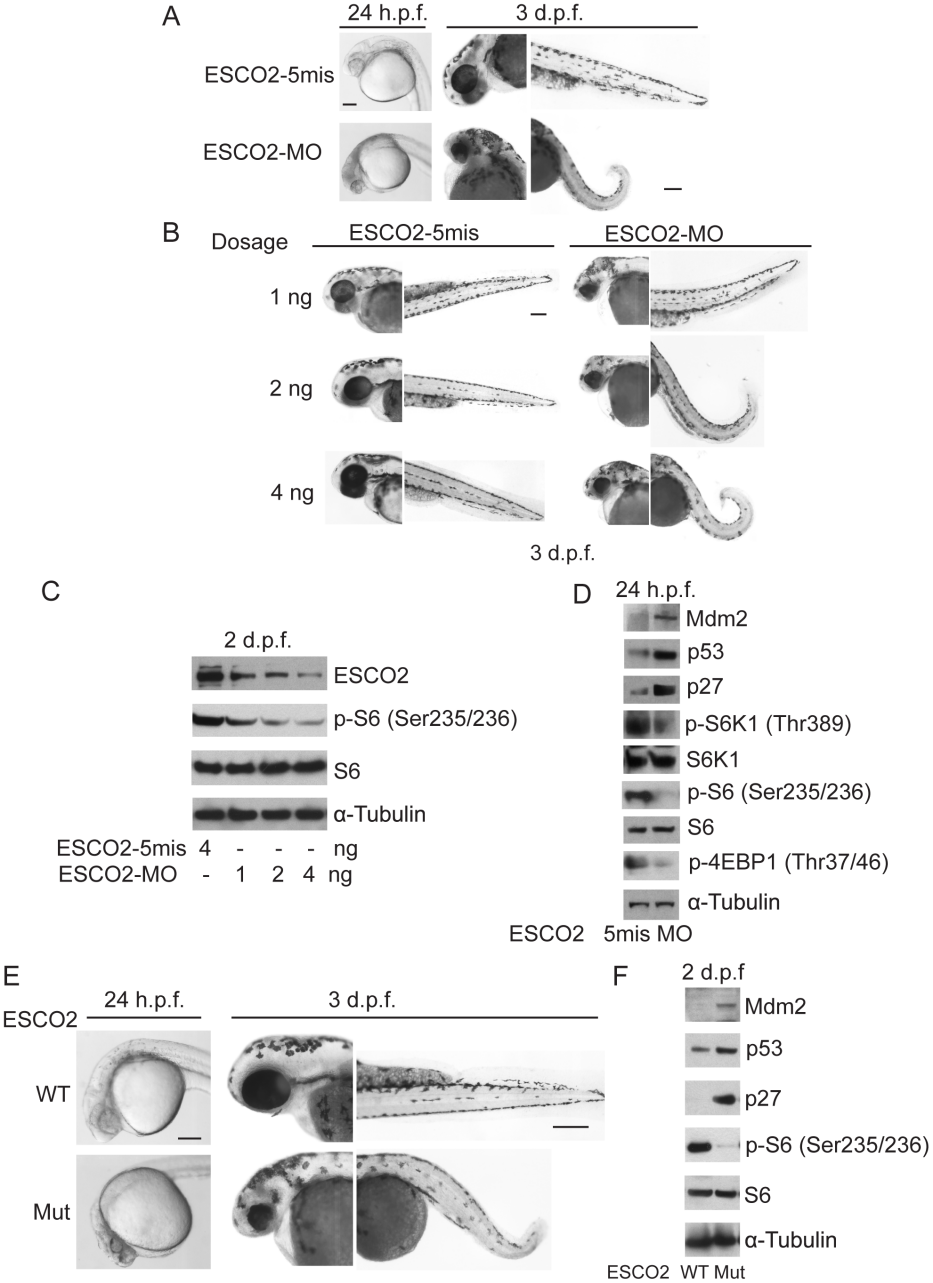
Reduced ESCO2 function is associated with mTOR inhibition, p53 activation, and dramatic developmental phenotypes in zebrafish. Total levels of S6, S6K1, and tubulin serve as loading controls. (A). Embryos were microinjected with 4 ng ESCO2-5mismatched morpholino (ESCO2-5mis) or ESCO2-ATG morpholino (ESCO2-MO) and photographed at 24 hours post fertilization (h.p.f.) and 3 days post fertilization (d.p.f.). Scale bar = 200 µm. (B). Embryos were microinjected with 1, 2, or 4 ng ESCO2-5mis or ESCO2-MO and photographed at 3 d.p.f. Scale bar = 200 µm. (C). Embryos were microinjected with 4 ng ESCO2-5mis or 1, 2, or 4 ng ESCO2-MO to test the effect of dosage on phophorylation of S6 by Western blot at 2 d.p.f. (D). ESCO2 morphant embryos (2 ng) show inhibition of the TOR pathway and accompanying activation of p53 at 24 h.p.f.. (E). ESCO2-transgenic mutant zebrafish embryos show gross developmental abnormalities compared with WT embryos at 24 h.p.f. and 3 d.p.f.. Bar = 200 µm. (F). ESCO2 mutant embryos show reduced S6 phosphorylation and upregulation of p53, Mdm2, and p27 by Western blot analysis.

To further examine the molecular etiology underlying the developmental defects in a whole animal model for RBS, we utilized both ESCO2 morphants and ESCO2 homozygous transgenic mutant zebrafish. Morphants (MO) were created by microinjecting zebrafish embryos (1–4 cells) with a morpholino to knockdown ESCO2 as previously described [42]. The control morpholino had 5-base mismatches (ESCO2-5mis). The ESCO2-5mis injected embryos did not show any phenotypic changes compared with uninjected WT embryos. As previously reported, both the ESCO2 morphant and mutant embryos had multiple developmental defects such as underdeveloped head and eyes, cardiac edema, short body length, curved tail, and loss of skin pigmentation (Figure 5A, B, E) [42], [77]. Western blot analysis showed similar findings with respect to the p53 and mTOR pathways as observed in human cells (Figure 5C, D, F). Furthermore, inhibition of the TOR pathway, as monitored by phosphorylation of S6, was found to increase in a dose dependent manner with the ESCO2 morpholino (Figure 5C). We used the ESCO2-splice morpholino (ESCO2-Splice MO), which shows a similar effect on embryo development as the ESCO2-ATG translation blocking morpholino (Figure S7A), to test ESCO2-mRNA rescue. The results showed that the defects of ESCO2-morphants and mutant embryos were markedly rescued following injection of in vitro transcribed RNA encoding ESCO2 (Figure S7A, B, C).

**Figure 6:**
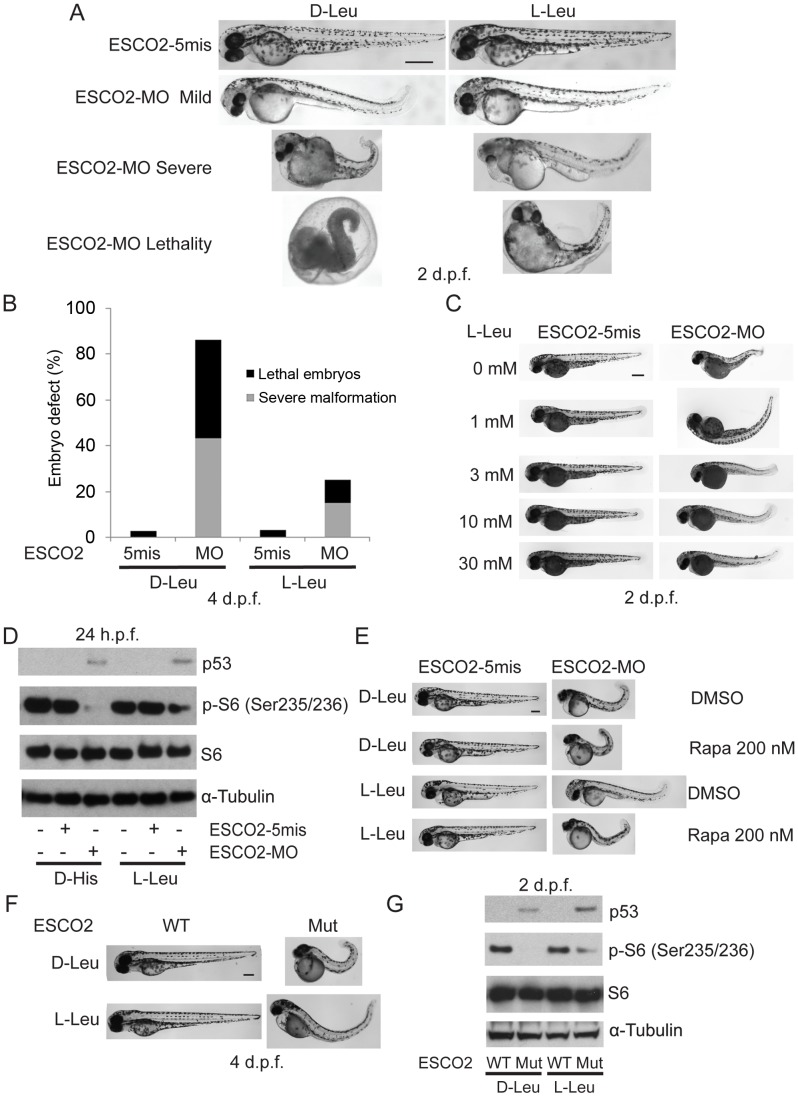
L-leucine partially improved developmental deficiencies of ESCO2 depleted embryos in a TOR pathway-dependent manner. (A). Embryos (1–2 cells) were injected with ESCO2-5mis or ESCO2-MO (4 ng) and immediately separated for D-Leu or L-Leu incubation (10 mM) for 2 d.p.f. L-Leu supplement partially rescued development of ESCO2-morphant embryos at a gross level. Scale bar = 200 µm. Animals were categorized as mildly affected, severely affected, or dead to further quantify the rescue. (B). The number of severely malformed and lethal embryos was quantified for ESCO2-depleted embryos in the presence of D-Leu or L-Leu supplement at 4 days post fertilization. A total of 73-115 embryos were quantified per condition (ESCO2-5mis with D-Leu (n = 73), ESCO2-MO with D-Leu (n = 114), ESCO2-5mis with L-Leu (n = 97), and ESCO2-MO with L-Leu (n = 115)). (C). Embryos (1–2 cells) were injected with ESCO2-MO (4 ng) and immediately transferred to L-Leu incubation at different concentrations. L-Leu supplement ameliorated the developmental defects of ESCO2 morphant embryos in a dosage-dependent manner. While the image is representative, about 20 embryos were analyzed per group. Bar = 200 µm. (D). Embryos (1–2 cells) were injected with ESCO2-5mis or ESCO2-MO (4 ng) and immediately separated into D-Leu or L-Leu incubation (3 mM) for 24 h.p.f.. By Western blot analysis, L-Leu supplement partially rescued phosphorylation of S6 in the ESCO2-MO embryos. S6 and tubulin serve as loading controls. Each sample contains ∼100 embryos. (E). Embryos (1–2 cells) were injected with ESCO2-5mis or ESCO2-MO (4 ng), and immediately separated into D-Leu or L-Leu incubation (10 mM) for 3 d.p.f., in the presence or absence of 200 nM rapamycin. While the image is representative, about 15 embryos were analyzed per group. Rapamycin curtails L-Leu rescue of ESCO2-morphants, and enhances malformation of ESCO2-depleted embryos. Bar = 200 µm. (F). WT and ESCO2 mutant embryos (1–2 cells) were incubated with 10 mM D-Leu or L-Leu for 4 d.p.f., and photographed. L-Leu treatment partially rescued development of ESCO2 mutant embryos. While the image is representative, about 10 embryos were analyzed per group. Bar = 200 µm. (G). WT and ESCO2 mutant embryos (1–2 cells) were incubated with 10 mM D-Leu or L-Leu for 2 d.p.f. Western blotting shows L-Leu treatment partially restored phosphorylation of S6 in ESCO2 mutant embryos, but p53 elevation persists. S6 and tubulin serve as loading controls.

To address whether the administration of L-Leu could rescue the phenotypic effects of ESCO2-defective zebrafish embryos, ESCO2 mutant embryos or ESCO2 morphants were treated with L-Leu, or D-Leu plus L-glutamine (L-Glu 4 mM) for 2 days post fertilization (d.p.f.). L-Glu has been demonstrated to promote the import of amino acids [36]. ESCO2-morphants and ESCO2-mutant embryos raised in egg water with the control amino acid D-Leu showed profound developmental failure compared with ESCO2-5mis control morphants or WT embryos (Figure 6A, C, E, F), including shortened body length and underdeveloped head and eyes. Interestingly, ESCO2 morphant and mutant embryos treated with L-Leu showed a remarkable improvement for all developmental deficiencies compared with those raised in egg water with D-Leu treatment (Figure 6A, B, C, E, F). To further test the cooperative effects of L-Glu and L-Leu, ESCO2-morphants were treated with L-Glu alone or L-Leu alone. Only the combination showed developmental rescue (Figure S7F), and this combination was used in all the experiments that follow. The effect was also specific to L-Glu and L-Leu since there was no improvement when morphants were treated with D-Leu, D-His, or L-Thr (Figure S7G). We assigned morphant embryos to one of three classes: 1) mildly affected, 2) severely affected, and 3) dead. L-Leu not only rescued severe malformation of ESCO2 morphants, but also promoted their survival (Figure 6A–B).

Next we tested whether L-Leu was rescuing development of ESCO2-morphants by stimulating TOR signaling. Embryos treated with L-Leu or D-His were collected and analyzed by immunoblot analysis. Phosphorylation of S6 is strongly reduced in ESCO2 morphant and mutant embryos. However, L-Leu partially restored phosphorylation of S6 in ESCO2 morphant and mutant embryos (Figure 6D, G). The ESCO2 mutant embryos were identified by fin clip and PCR genotyping analysis. Rapamycin treatment enhanced the defects observed in ESCO2 morphants at a concentration that did not affect WT embryos. Consistent with L-Leu acting through mTOR, rapamycin curtailed the rescue by L-Leu in ESCO2 morphants (Figure 6E), but did not curtail the rescue by ESCO2 mRNA (Figure S7C). The data indicate L-Leu stimulation of the TORC1 pathway can improve development and survival in ESCO2 defective embryos.

### L-leucine partially rescues craniofacial defects, cell survival, and proliferation in ESCO2 defective embryos

**Figure 7:**
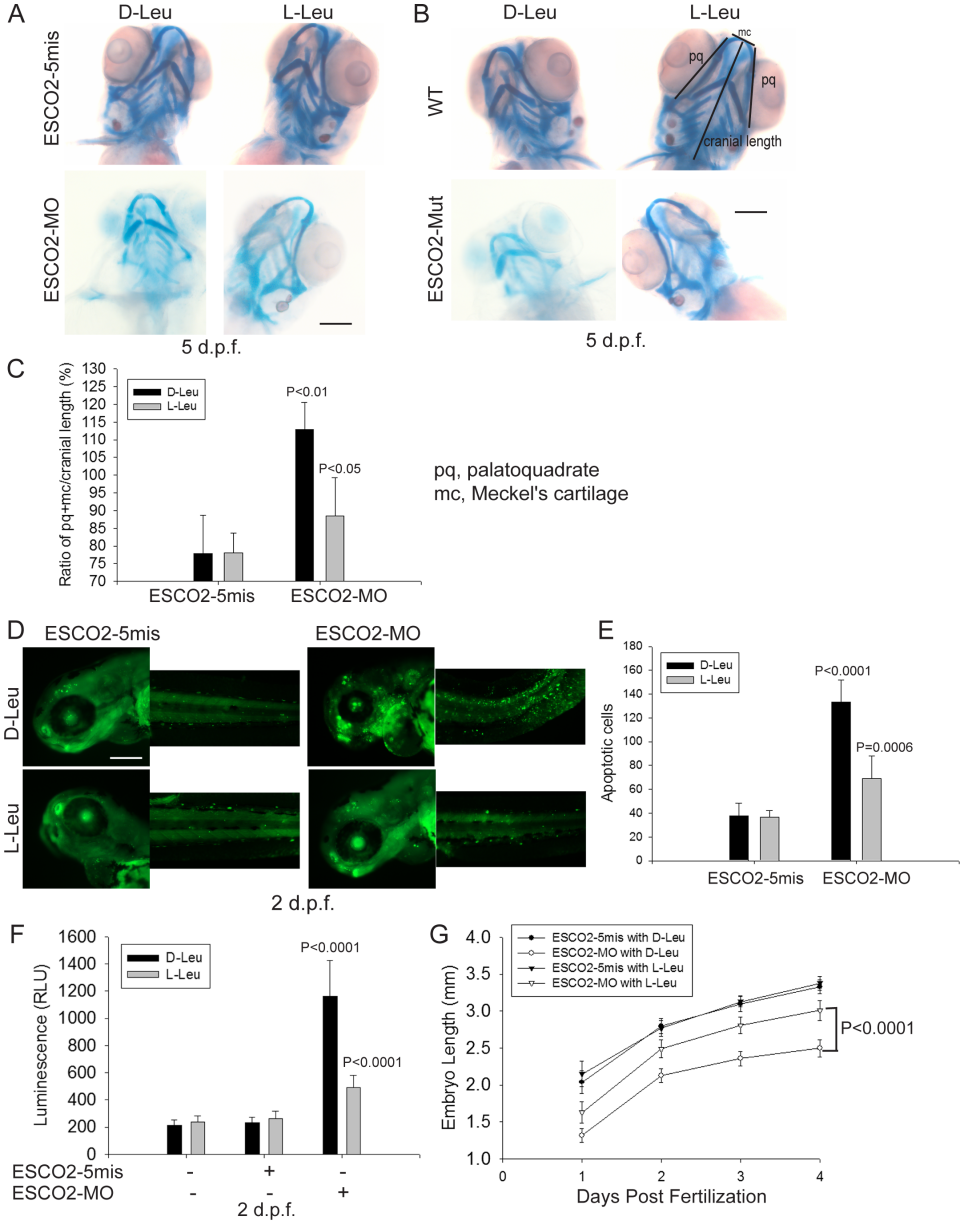
L-leucine rescues many aspects of development in ESCO2-depleted embryos. (A). Embryos (1–2 cells) were injected with ESCO2-5mis or ESCO2-MO (4 ng), and immediately separated into D-Leu or L-Leu incubation (10 mM). After 5 d.p.f., the embryos were stained with Alcian blue to detect cartilage development. Scale bar = 200 µm. While the image is representative, about 15 embryos were analyzed per group. (B). WT or ESCO2 mutant embryos were treated with D-Leu or L-Leu (10 mM). After 5 d.p.f., the embryos were stained with Alcian blue to detect cartilage development. While the image is representative, about 10 embryos were analyzed per group. L-leucine partially rescued head size and cartilage formation in (A) and (B). (C). Cranial development was quantified for the data in (A) using the sum of the pq (palatoquadrate) cartilage and mc (Meckel's cartilage) divided by cranial length, as indicated with lines in (B). The measurement was done on 3 embryos per group. P<0.01, ESCO2-MO with D-Leu treatment *vs* ESCO2-5mis with D-Leu treatment; P<0.05, ESCO2-MO with L-Leu treatment *vs* ESCO2-MO with D-Leu treatment. (D). Embryos (1–2 cells) were injected with ESCO2-5mis or ESCO2-MO (2 ng), and immediately separated for D-Leu or L-Leu incubation (10 mM). After 2 d.p.f., the embryos were stained with acridine orange to detect apoptotic cells. Scale bar = 200 µm. (E). The number of apoptotic cells was quantified. P<0.0001, ESCO2-MO with D-Leu treatment *vs* ESCO2-5mis with D-Leu treatment; P = 0.0006, ESCO2-MO with L-Leu treatment *vs* ESCO2-MO with D-Leu treatment. (F). Embryos (1–2 cells) were treated as in (A). After 2 d.p.f., single cell suspensions of 10 embryos were generated in triplicate by mashing and filtering in PBS/10% NCS through cell strainers (100 µm, BD Falcon). Cells were resuspended in PBS/10% NCS and suspensions used in Caspase-Glo 3/7 Assays (Promega) according to the manufacturer's instructions. Luminescence was measured after 75 minutes on a multi-detection microplate reader. L-Leu treatment suppressed caspase 3/7 activation in the ESCO2-MO embryos. P<0.0001, ESCO2-5mis with D-Leu *vs* ESCO2-MO with D-Leu; P<0.0001, ESCO2-MO with L-Leu *vs* ESCO2-MO with D-Leu. (G). Embryos (1–2 cells) were treated as in (A). Embryo length was measured every day up to 4 d.p.f. with a mini-ruler under a Leica Stereoscope for ESCO2-MO and 5-mismatched controls. L-Leu treated ESCO2 morphants had a significantly longer body length compared with ESCO2 morphants treated with D-Leu (P<0.0001, 2-way ANOVA). The measurements were performed from head to tail for 10 embryos per group.

To further study the effect of L-Leu on development of ESCO2 defective embryos, we assessed cartilage formation by staining with alcian blue at 5 days post fertilization (d.p.f.). We observed marked abnormalities in the craniofacial elements in ESCO2 morphant and mutant embryos compared with control embryos (Figure 7A–B). The effect was quantified using the relative ratio of the sum of the pq (palatoquadrate) cartilage and mc (Meckel's cartilage) divided by cranial length, based on a method developed in a recent report [78] (Figure 7C). Impaired mTOR activity via Akt knockout has previously been shown to result in delayed skeletal development and poor cartilage matrix in mice [79], [80]. With L-Leu treatment, cartilage length and head size were markedly recovered (Figure 7A–C).

ESCO2 morphants had previously been reported to show a global increase in apoptotic cells by TUNEL assay and acute elevation of caspase3/7 activity [42]. We were able to recapitulate these findings in ESCO2 morphant embryos using acridine orange staining of apoptotic cells (Figure 7D–E) and an assay for caspase3/7 activity (Figure 7F). The cell death was reported to be p53 independent because a p53 mutation failed to rescue the observed apoptosis [42]. We also observed that knockdown of p53 levels had only a small rescue effect on ESCO2 deficient embryos (Figure S8A–C). p53 knockdown did not significantly suppress caspase 3/7 activation in the ESCO2 morphants (Figure S8D). While L-Leu significantly rescued cranial length, p53 knockdown did not (Figure S8E–F). Since L-Leu significantly suppressed apoptosis levels in ESCO2 depleted embryos and caspase 3/7 activation, apoptosis may be coupled to regulation of the mTOR pathway. Measurements of embryo length showed that L-Leu partially restored body growth in ESCO2 morphant embryos (Figure 7G).

**Figure 8:**
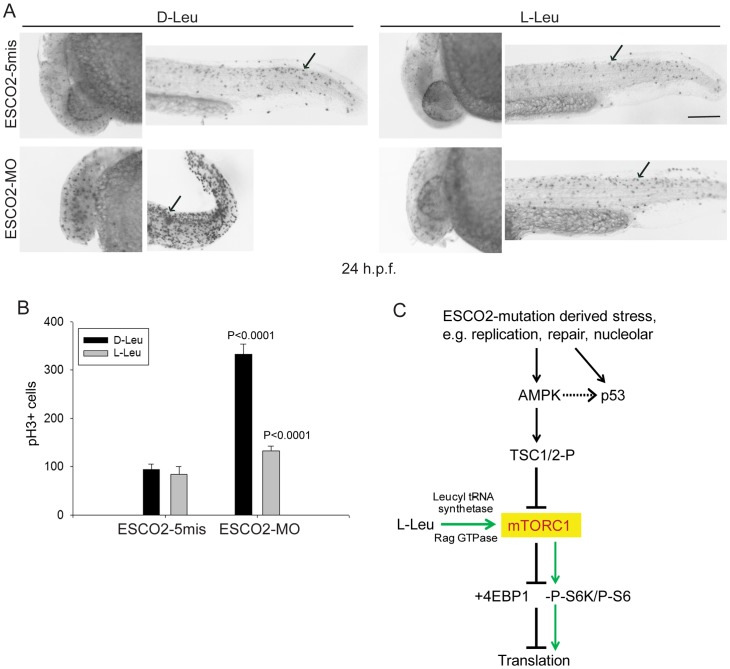
ESCO2 depletion in zebrafish is associated with an increase in phospho-H3 staining, and L-leucine partially rescues the increase. (A). Embryos (1–2 cells) were injected with ESCO2-5mis or ESCO2-MO (4 ng), and immediately separated for D-Leu or L-Leu incubation (10 mM). At 24 h.p.f., cells were immunostained with anti-phospho-Histone H3 (pH3) antibody to detect mitotic cells. Scale bar = 200 µm. (B). The number of cells in mitosis was quantified for 5 embryos per group. P<0.0001, ESCO2-MO with D-Leu treatment *vs* ESCO2-5mis with D-Leu treatment; P<0.0001, ESCO2-MO with D-Leu treatment *vs* ESCO2-MO with L-Leu treatment. (C). A working model for the pathways involved in RBS is presented. Due to mutation in ESCO2, significant intracellular stress occurs due to defects in DNA replication, repair, and rDNA processes. This stress is detected by AMPK which can signal the activation of p53 [115], [116] and the phosphorylation of TSC2. The phosphorylation of TSC2 will act to inhibit mTORC1 and downstream effectors such as 4EBP1, S6, and S6 kinase with the ultimate effect being the inhibition of translation. With the addition of L-leucine (green arrows), the leucyl tRNA synthetase will collaborate with the Rag GTPase to activate mTORC1, partially rescuing translation.

ESCO2 depleted zebrafish embryos exhibit a cell cycle block in G2/M phase [42]. Given our results in human RBS cells, we wondered if the mitotic delay could be overcome by L-Leu supplementation during embryo growth. The ESCO2 morphants were immunostained with phospho-Histone H3 (pH3) in the presence of D-Leu or L-Leu, and the pH3 positive cells were quantified. Mitotic cells were robustly elevated in ESCO2 depleted embryos, but L-Leu treatment significantly reduced their number (Figure 8A–B). Knockdown of p53 had a similar, but milder, effect (Figure S9).

## Discussion

The cohesinopathies have been proposed to arise from defects in transcription during embryogenesis. However, the mechanism by which mutations in the cohesin ring or cohesin associated factors caused these transcriptional defects has proven elusive. Our results demonstrate the activation of p53 and inhibition of mTOR in RBS. Furthermore, the defect in protein translation in RBS can be targeted via stimulation of the TOR pathway with L-Leu. Although p53 activation and nucleolar fragmentation persist with L-Leu treatment, L-Leu boosts TOR function which can then increase protein synthesis and cell proliferation. This “band-aid” is sufficient at the organismal level to partially rescue development (see working model, Figure 8C). In contrast, p53 inhibition partially rescues cell division, but has less effect on cell death, protein synthesis, rRNA production, and developmental phenotypes in zebrafish. Our study represents the first RBS preclinical animal model in which L-Leu supplement produces an improvement in the developmental defects associated with RBS. ESCO2-inactivated mice showed termination of embryogenesis in the pre-implantation period due to a prometaphase delay and loss of cell viability [81]; in the future it would be interesting to test whether L-Leu could rescue embryogenesis in ESCO2-inactivated mice. Our study suggests some of the differential gene expression during embryogenesis in RBS may be due to translational defects.

While RBS is rare, Cornelia de Lange syndrome (CdLS) is a more common cohesinopathy (1 in 10,000 births), and is also associated with mutations in cohesin genes. Depletion of cohesin genes (Rad21, Smc3, Smc1) is associated with upregulation of p53 in zebrafish morphants [42], [82], [83], [84]. Smc3 and Smc1 are mutated in CdLS [85] and Rad21 is mutated in a related cohesinopathy [86]. It will be important to explore whether p53 activation in these cases is due in part to nucleolar stress and defects in ribosome biogenesis. A CdLS mutation in Smc1 in yeast resulted in reduced rRNA, protein synthesis, and actively translating ribosomes [87], demonstrating ribosome defects can be associated with Smc1 mutation. A CdLS mouse model (NIPBL+/−) showed decreased spontaneous adipogenesis and adipocyte differentiation as well as reduced body fat [88]. While p53 mRNA is not upregulated in zebrafish morphants for NIPBL [89], the mTORC1-S6K1 pathway is needed for commitment to adipogenesis and the generation of de novo adipocytes [90]. Therefore, inhibition of the TOR pathway could potentially contribute to the lean body habitus of the NIPBL+/− mouse. In addition, cohesin binds to the c-Myc gene locus [82], [91], [92], [93], [94], and c-Myc gene expression is downregulated in various CdLS cells with SMC1A and SMC3 mutations and a zebrafish CdLS model with NIPBL deficiency [83], [95], [96]. Since c-Myc serves as a direct positive regulator of ribosome biogenesis and protein synthesis [97], we speculate that CdLS may also be associated with reduced ribosome and protein biosynthesis. It will be important in the future to determine which mutations in cohesin are associated with nucleolar stress, inhibition of the TOR pathway, and reduced translation.

There are several disorders associated with defects in ribosome biogenesis, including Treacher Collins syndrome [98], 5q-syndrome [99], Diamond-Blackfan anemia (DBA) [100], Shwachman-Bodian-Diamond syndrome (SBDS) [101], and dyskeratosis congenita [102]. There is evidence for involvement of p53 and mTOR in these ribosomopathies. In all of these diseases p53 is upregulated, possibly due to nucleolar stress [103], [104], [105], [106]. Some developmental phenotypes are shared in common between RBS and these disorders, including craniofacial, cardiac, urogenital, and limb defects [45], [106], [107]. The craniofacial dysmorphology of Treacher Collins syndrome, which can be caused by mutation in the nucleolar phosphoprotein Tcof, has been modeled in mice. The developmental defects in Tcof mice can be partially rescued by inhibition of p53 function [106]. Gross developmental phenotypes associated with mouse models for DBA and 5q-syndrome are rescued by loss of p53 function [45], [108], but anemia is not [109]. In contrast, p53 inhibition showed weak rescue in a zebrafish model for SBDS [110], more similar to the observations for the zebrafish model for RBS, suggesting both p53 dependent and independent pathology for ribosome biogenesis defects. Interestingly, the amino acid L-leucine ameliorates developmental defects and anemia associated with mouse and zebrafish models for DBA and 5q-Sydrome, through the mTOR pathway [111], [112]. We propose that RBS may share common underlying physiology with other ribosomopathies, with similar pathways being affected. Future work will reveal whether manipulating the p53 and TOR pathways will provide rescue for other cohesinopathies and ribosomopathies.

In summary, we demonstrate for the first time in patient cells and an animal model of RBS that the TOR pathway is strongly impaired. Furthermore, L-leucine treatment of ESCO2 deficient zebrafish embryos and human cells activates the TOR pathway and relieves proliferation and developmental defects linked to RBS phenotypes. Some of the transcriptional changes that occur in RBS may occur as a result of translational defects. Our observations support the hypothesis that RBS may be partially attributed to defects in translation.

## Materials and Methods

### Ethics statement

All animals were handled in strict accordance with good animal practice as defined by the relevant national and/or local animal welfare bodies, and all animal work was approved by the Stowers Institute for Medical Research, Institutional Animal Care and Use Committee.

### Zebrafish line maintenance

Zebrafish (AB strain) and esco2-transgenic mutant line were maintained by Reptile & Aquatics facility at the Stowers Institute for Medical Research as described previously [113]. Genotyping is shown in Figure S10.

ESCO2 genotyping primer-1, 5′ GTACTATTCTACCCGGTAAGTGG 3′

ESCO2 genotyping primer-2, 5′ GACGAGCTAATCTGCAGTTCAAG 3′

ESCO2 genotyping primer-3, 5′ GCCAAACCTACAGGTGGGGTC 3′

### Cell culture

Human RBS fibroblasts were a gift from Johan P. de Winter [41]. Briefly, primary skin fibroblasts from a two-month old male RBS patient homozygous for the mutation 877_878 delAG in exon 4, were immortalized by transfection with a plasmid encoding the SV40 large-T antigen. Several weeks after transfection colonies of transformed cells appeared, which were mixed and further propagated. The transformed cells have been in continuous culture for over 60 passages and were therefore considered immortal. This immortalized RBS cell line (VU1199-F SV40), primary RBS fibroblasts (VU1174-F), and wild type cell lines (SV40) were cultured in Ham's F10 medium (Gibco, Paisley, UK) supplemented with 10% fetal bovine serum (FBS, Hyclone, Logan, USA). Stable cell lines were generated by transfection of *Pvu*I linearized expression vector pIRESneo containing cDNAs encoding V5-ESCO2. These stable cell lines were cultured in complete medium containing G418 at 150 µg/ml (Invitrogen, U.S.A). Cells were trypsinized with 0.05% Trypsin-EDTA (Invitrogen, Grand Island, NY, USA), sub-cultured, and maintained in a humid incubator (37°C, 5% CO_2_). Other RBS cells (GM21873 and GM21872) were purchased from Coriell Institute for Medical Research. GM21872 is an untransformed skin fibroblast line from a 25 weeks female fetal RBS patient. These cells were cultured in Dulbecco's Modified Eagle Medium plus 15% fetal bovine serum. GM21873 is untransformed amniotic fluid-derived cell line from 20 weeks old female fetal RBS patient. These cells were cultured with AmnioMAX II Complete Medium. Both GM21872 and GM21873 are mutant for ESCO2. The control cells were GM00957 and I91S-05, normal human amniotic fluid-derived cells and untransformed fetal skin fibroblasts, respectively.

### SiRNA transfection

Human RBS cells were transfected with p53-siRNA (#6231, *Cell Signaling Technology, Inc.*) or control siRNA-A (sc-37007, *Santa Cruz Biotechnology, Inc.*) using siPORT NeoFX Transfection Agent (*Ambion | Life Technologies, Inc.*) following the manufacturer's instructions. 24 hours after transfection, the cells were harvested and subjected to Western blot analysis.

### Microinjection of zebrafish embryo

Antisense morpholino oligonucleotides (MOs) were obtained from GeneTools, LLC based on Mönnich M *et al* [42]. For microinjection, 2 nL of morpholino solution diluted in Danieau's buffer was injected into the yolk of wild type embryos at the 1 to 2-cell stage. Morpholino sequences and effective amounts were ESCO2-ATG-MO, 5′-CTCTTTCGGGATAACATCTTCAATC-3′ (1 ng–4 ng), and ESCO2-5mis-MO, 5′-GTAAACTACACAATGTTACCTCTCG-3′ (1 ng–10 ng), and ESCO2-Splice-MO, 5′-GTAAACTACACAATGTTACCTCTCG-3′(6 ng–10 ng). ESCO2-ATG-MO targets the ATG start codon, and ESCO2-Splice-MO targets the 5′ donor of the exon/intron boundary of intron 2 of ESCO2, to create knockdown “morphant” embryos.

p53-morpholino, 5′-GCGCCATTGCTTTGCAAGAATTG-3′ (1 ng–4 ng)

Control morpholino is a Random Control MO produced by GeneTools, LLC.

Full-length zebrafish ESCO2 mRNA was subcloned into pCS2+. The ESCO2 mRNAs for microinjection were generated using a mMessage mMachine Kit (Ambion) by in vitro transcription, and 200 pg of each was injected into ESCO2 morphants and the mutant embryos at the 1-cell stage.

Primers:

ESCO2 full length mRNA primer-F: 5′ CGGGATCCCGATGTTATCCCGAAAGAGAAAAC 3′

ESCO2 full length mRNA primer-R: 5′ CCATCGATGGTTAGCTGATGAAGTTGTACACC 3′

Control mRNA primer-F: 5′ CGGGATCCCGATGTGTGACGACGACGAGACT 3′

Control mRNA primer-R: 5′ CCATCGATGGTTAGAAGCACTTGCGGTGGA 3′

### SDS PAGE and Western blot analysis

Sodium dodecyl sulfate-polyacrylamide gel electrophoresis (SDS-PAGE) was performed using NuPAGE Novex 4%–12% Bis-Tris precast gels (Invitrogen). Western blotting was performed according to standard protocol using a nitrocellulose (Whatman, Protran) or PVDF (Millipore, Immobilon-P) membrane. The following antibodies were used: phospho-S6K1 (Thr389), S6K1, phospho-S6 (Ser235/236), 4EBP1, p53, phospho-AMPKα, AMPKα, phospho-Acetyl-CoA Carboxylase (p-ACC), Acetyl-CoA Carboxylase (ACC), phospho-Tuberin/TSC2, Tuberin/TSC2 (Cell Signaling Technology, Beverly, MA, USA), α-EFO2 (ESCO2), S6 (Santa Cruz Biotechnology, U.S.A), α-tubulin (Sigma, MO). Secondary antibodies were HRP linked, anti-rabbit IgG (from donkey) and anti-mouse IgG (from sheep), (GE Healthcare, NA934V, NA931V, and NA935V, respectively). In Figures 2 and 4, the quantitative analysis of protein expression was performed by ImageQuant TL software.

### Metabolic labeling-protein of RBS cells

Cultured wild-type (WT), ESCO2-mutant and ESCO2-corrected human RBS fibroblasts, two pairs of normal and RBS cells were grown in leucine-free Dulbecco's modied Eagle's medium (DMEM) plus 10% FBS. Before labeling, the cells were incubated with indicated L-Leu or D-Leu for 24 hrs. Cells were then washed in PBS twice, switched to 3 mL Met/Cys-free DMEM containing 10 µM MG-132, a proteasome inhibitor. and pulsed with 30 µCi of ^35^S-methionine for the indicated time (4 hrs). Cells were lysed in RIPA buffer (50 mM Tris, pH 7.2; 150 mM NaCl; 1% sodium deoxycholate; 0.1% SDS; 1% Triton-X 100; 10 mM NaF; 1 mM Na3VO4). Proteins were precipitated by the addition of hot 10% trichloroacetic acid. After centrifugation, the precipitate was washed twice in acetone. The precipitate was dissolved in 100 µL of 1% SDS and heated at 95°C for 10 min. An aliquot of the SDS extract was counted in Ecoscint for ^35^S radioactivity in a liquid scintillation spectrometer to determine the amount of ^35^S-methionine incorporated into proteins.

### Metabolic labeling-rRNA of RBS cells

Methods for rRNA labeling were derived from a previous report [114]. Cells were grown as for protein labeling. ^3^H-uridine (5 µCi) was then incubated with 10^6^ cells from each group for two hours. Total RNA was isolated with TriZol reagent (Invitrogen, U.S.A) and the concentration of each RNA sample was measured by OD_260/280_. 1 µg of each sample was counted in a Beckman LS 6500 multipurpose scintillation counter to determine the amount of ^3^H-uridine incorporated. Three independent cultures were labeled to derive the standard deviation. Significance relative to WT was calculated using an unpaired t test.

### Immunofluorescence

Cells seeded on coverslips were washed in PBS, fixed for 10 min at room temperature 20–22°C with 4% paraformaldehyde, permeabilized for 5 min in PBS containing 0.5% Triton X-100 and washed in PBS. After blocking for 30 min in PBS containing 1% BSA at room temperature, the preparations were incubated overnight at 4°C with the following antibodies diluted in PBS containing 1% BSA: mouse anti-fibrillarin with 1∶500 dilution, rabbit anti-nucleolin (H-250; Santa Cruz) with 1∶500 dilution. The coverslips were then washed in PBS and incubated for 30 min at room temperature with the secondary antibodies Alexa Fluor 488 goat anti-mouse and Alexa Fluor 555 donkey anti-rabbit (Molecular Probes) diluted in PBS containing 1% BSA and then washed in PBS. The coverslips were mounted on slides and analyzed by fluorescence imaging with a Zeiss Axioplan II confocal microscope.

### Cell proliferation and survival assay

For p53 inhibitor (Pifα) treatment, cells were seeded in DMEM supplemented with 10% FBS in 6-well plates at a density of 5×10^4^ cells/well (in triplicate) and grown overnight at 37°C in a humidified incubator with 5% CO2. Treatments added the next day included Pifα (10 µM), DMSO, L-Leu, or D-Leu. Cells were trypsinized and stained with trypan blue, and the number of viable cells and dead cells were quantified using a Brightline hemocytometer.

### Cell cycle analysis of human RBS cells with flow cytometry

Human RBS cells were cultured and 3×10^6^ cells for each sample was used for analysis. Cells were washed in PBS, then fixed with the addition of 1 mL of 70% cold at room temperature for 30 minutes. After washing with PBS, the cells were incubated with Rnase A and propidium iodide staining solution for 2 hrs. Samples were analyzed by flow cytometry. Data analysis was performed using FlowJo Version 7.6.4 software.

### Alcian blue staining for cartilage

Embryos were collected and most of the liquid was removed. 1 ml 2%PFA/1XPBS pH 7.5 was added to the embryos followed by nutation for 1 hr. Embryos were washed 1×10 mins with 100 mM Tris pH 7.5/10 mM MgCl_2_, followed by addition of 1 ml of 0.04% alcian/10 mM MgCl_2_ stain pH 7.5 and overnight incubation with nutation. Embryos were washed with 80% ETOH/100 mM Tris pH 7.5/10 mM MgCl2 for 5 mins, then 50% ETOH/100 mM Tris pH 7.5 for 5 mins, then 25% ETOH/100 mM Tris pH 7.5 for 5 mins. 1 ml 3%H202/0.5%KOH was added. Open tubes were incubated for 10 mins, followed by 2×10 mins with 1 ml 25% glycerol/0.1% KOH. Bleach was rinsed out and 1 ml 50% glycerol/0.1%KOH was added. Embryos were mutated 10 mins followed by a wash with fresh 50% glycerol/0.1%KOH.

### Cell cycle analysis and immunohistochemistry

Dechorionated embryos with or without ESCO2-knockdown were stained with phosphorylated histone H3 (pH3) to measure mitotic cells in G2/M stage at 24 h.p.f. Phospho-Histone H3 was detected using an anti-Phospho-Histone H3 (Ser10) antibody (Cell Signaling), followed by detection with anti-rabbit-HRP (Sigma). The number of pH3-staining positive cells was quantified on a consistent field (end of yolk extension to end of tail in stage-matched embryos) for 5 embryos per group. To compare cell counts between the samples, a two-sided t-test was used.

### Apoptosis analysis of zebrafish embryos

The morphants were stained with acridine orange at 2 d.p.f. to detect apoptotic cells, which show green fluorescent granulated spots. The number of apoptotic cells was quantified on a consistent field (end of yolk extension to end of tail in stage-matched embryos) for 5 embryos per group. To compare cell counts between the samples, a two-sided t-test was used.

### Imaging

Stained embryos were fixed in 4% PFA, and preserved and imaged in 90% glycerol. Live morphants were anesthetized in 0.4% tricaine in egg water for 3 minutes and immobilized in 1.5% methylcellulose. Embryos were visualized with a Leica Stereoscope (Leica MZFLIII or Leica MZ16FA), a Leica DFC310FX camera and Leica application suite software. Images were processed using Photoshop CS5 (Adobe). Embryo body length was measured at low magnification (3.2X–6.3X) using a microscope ruler with Leica microsystems. The measurements were analyzed using a 2-way ANOVA with Bonferronis posttest analysis to assess the effects of ESCO2-knockdown, L-leucine, and time in combination. In addition, severely deformed and dead embryos were counted and statistically analyzed using a 2-way ANOVA one-way model to compare the differences.

### RNA Isolation from zebrafish embryos by Trizol purification

30–50 embryos were homogenized in 1 mL of Trizol with a 20-gauge needle and syringe. The sample volume did not exceed 10% of total volume. Homogenized samples were incubated for 5 minutes at RT (room temperature). 200 µL chloroform was added followed by a brief vortex and ∼3 minute incubation at room temperature. The sample was centrifuged at 12000 g for 10 minutes at 4°C. Following centrifugation, the aqueous phase was transferred to a fresh tube. An equal volume of isopropyl alcohol (2-propanol) was added to the sample followed by mixing and incubation for 10 minutes at room temperature. The sample was centrifuged at 12000 g for 10 minutes at 4°C. The supernatant was removed and discarded. 1 mL 75% Ethanol (RNase free) was added to the sample. The sample was centrifuged at 12000 g for 10 minutes at 4°C. The supernatant was removed and discarded. The pellet was briefly air dried and then resuspended in 10 µL RNase-free water. Samples were stored at −80°C.

### Halo assays

An overnight culture of yeast was grown, followed by dilution to OD_600_∼0.1. 1 mL of the diluted culture was spread onto a plate. Excess culture was removed and the plates were allowed to dry for ∼1 hr. 10 ul of rapamycin at the desired concentration was spotted onto sterile filter disks (DIFCO concentration disks, 1/4″ diameter). With sterile tweezers, the disk was placed onto the lawn of cells. Plates were incubated for 1–2 days. The diameter of each halo (region of no cell growth) around the filter disk was measured.

### Measurement of intracellular reactive species oxygen

Cells were incubated with 10 µM of carboxy-H2DCFDA (C400, Invitrogen Corporation) in the culture medium at 37°C for 3 hours, and the resulting fluorescence measured with a Synergy HT Multidetection Microplate Reader at an excitation wavelength of 485/10 nm and an emission wavelength of 528/20 nm.

### Statistical analysis

The results are reported as mean values±standard error (mean±s.e.). Statistical analysis was performed by Student's t-test with SigmaPlot-Systat Software (Sigmaplot Software Inc). An ANOVA two-way model was used to compare continuous variables. A *P* value <0.05 was considered statistically significant.

## Supporting Information

Figure S1 Inhibition of p53 did not rescue rRNA production or protein synthesis in human RBS cells. (A). The immortal WT, ESCO2-mutant and corrected cells were treated with p53 inhibitor Pifα (10 µM) or DMSO for 24 hrs, and FACScan analysis was performed with cell cycle measurements. (B). Untransformed WT and RBS fibroblasts were cultured in DMEM plus 10% FBS, in the presence of Pifα (10 µM) or DMSO for 24 hrs. ^3^H-uridine labeling experiments showed that rRNA production of RBS cells was not rescued by Pifα treatment. Each bar represents the average ± SEM of the ratio of the measurement of the indicated ^3^H-uridine incorporation into rRNA, as calculated for three independent samples. P = 0.156, ESCO2-Mut+Pifα *vs* ESCO2-Mut+DMSO. (C). Untransformed WT and RBS fibroblasts were cultured in DMEM plus 10% FBS, in the presence of Pifα (10 µM) or DMSO for 24 hrs. ^35^S-methionine labeling showed that protein synthesis in RBS cells was not rescued by Pifα treatment. Each bar represents the average ± SEM of the ratio of the measurement of the indicated ^35^S-methionine incorporation, as calculated for three independent samples. P = 0.084, ESCO2-Mut+Pifα *vs* ESCO2-Mut+DMSO.
(TIF)

Figure S2 Nucleolar fragmentation was not rescued by inhibition of p53 in RBS cells. WT, RBS and corrected RBS cells were cultured in DMEM plus 10% FBS with p53 inhibitor Pifα (10 µM) or DMSO for 24 hrs. Cells were immunostained with anti-fibrillarin antibody, and imaged with confocal microscopy. DNA was stained with DAPI. The quantification of the nucleolar area was performed as in Figure 3. Bar = 10 µm. About 20 cells were quantified for each sample.
(TIF)

Figure S3 The *eco1-W216G* yeast mutant is hypersensitive to rapamycin. (A). Cultures of the indicated genotype were spread onto a plate and discs with the indicated amount of rapamycin were placed on the plate. The zone in which the yeast do not grow, or “halo,” showed that growth of the *eco1-W216G* mutant was hypersensitive to rapamycin. The diameter of each halo was measured at 1 day, and the growth of *eco1-W216G* mutant cells was reduced by about 50% compared with WT cells. The *tor1* mutant was used as a control for a strain that is not sensitive to rapamycin. (B). The experiment in (A) was repeated and the halo was measured at 2 days.
(TIF)

Figure S4 L-leucine rescues G2/M delay, rRNA production, and protein synthesis in RBS cells. (A). The immortal WT, ESCO2-mutant and corrected cells were cultured in the presence of basal levels of L-leucine (0.8 mM) or high levels of L-leucine (3 mM) for 24 hrs, and FACScan analysis with cell cycle measurement was performed. (B). WT and RBS cells were cultured in leucine-free DMEM plus 10% FBS, and supplemented with D-Leu or L-Leu (10 mM) for 24 hrs. ^3^H-uridine labeling showed that L-Leu partially rescued rRNA production in RBS cells. Each bar represents the average ± SEM of the ratio of the measurement of the indicated ^3^H-uridine incorporation into rRNA, as calculated for three independent samples. P = 0.0004, HSF ESCO2-Mut+D-Leu *vs* HSF ESCO2-WT+D-Leu; P = 0.0001, AFC ESCO2-Mut+D-Leu *vs* AFC ESCO2-WT+D-Leu; P = 0.0082, HSF ESCO2-Mut+L-Leu *vs* HSF ESCO2-Mut+D-Leu; P = 0.0003, AFC ESCO2-Mut+L-Leu *vs* AFC ESCO2-Mut+D-Leu. (C). WT and RBS cells were cultured as in (A) with or without 100 nM rapamycin. ^3^H-uridine labeling experiments showed that L-Leu partially rescued rRNA production of RBS cells via a mTORC1-dependent pathway. P = 0.0008, ESCO2-Mut+D-Leu with DMSO *vs* ESCO2-WT+D-Leu with DMSO; P = 0.0995, ESCO2-Mut+D-Leu with DMSO *vs* ESCO2-WT+D-Leu with rapamycin; P = 0.013, ESCO2-Mut+L-Leu with DMSO *vs* ESCO2-Mut+D-Leu with DMSO; P = 0.3982, ESCO2-Mut+L-Leu with rapamycin *vs* ESCO2-Mut+D-Leu with DMSO. (D). WT and RBS cells were cultured as in (A). ^35^S-methionine labeling showed that L-Leu partially rescued protein synthesis in RBS cells. Each bar represents the average ± SEM of the ratio of the measurement of the indicated ^35^S-methionine incorporation, as calculated for three independent samples. P = 0.0002, HSF ESCO2-Mut+D-Leu *vs* HSF ESCO2-WT+D-Leu; P<0.0001, AFC ESCO2-Mut+D-Leu *vs* AFC ESCO2-WT+D-Leu; P = 0.0028, HSF ESCO2-Mut+L-Leu *vs* HSF ESCO2-Mut+D-Leu; P = 0.001, AFC ESCO2-Mut+L-Leu *vs* AFC ESCO2-Mut+D-Leu. (E). WT and RBS cells were cultured as in (B). ^35^S-methionine labeling experiments showed that L-Leu partially rescued protein synthesis RBS cells via a mTORC1-dependent pathway. P = 0.0007, ESCO2-Mut+D-Leu with DMSO *vs* ESCO2-WT+D-Leu with DMSO; P = 0.0035, ESCO2-Mut+D-Leu with DMSO *vs* ESCO2-WT+D-Leu with rapamycin; P = 0.0035, ESCO2-Mut+L-Leu with DMSO *vs* ESCO2-Mut+D-Leu with DMSO; P = 0.5412, ESCO2-Mut+L-Leu with rapamycin *vs* ESCO2-Mut+D-Leu with DMSO.
(TIF)

Figure S5 Nucleolar organization was not rescued by L-leucine treatment in immortalized RBS cells. WT, RBS and corrected RBS cells were cultured in DMEM plus 10% FBS with basal levels of L-Leu (0.8 mM in regular medium) or high levels of L-Leu (3 mM) for 2 days. Cells were immunostained with anti-fibrillarin antibody, and imaged with confocal microscopy. DNA was stained with DAPI. The quantification of the nucleolar area was performed as in Figure 3. Bar = 10 µm. About 20 cells were quantified for each sample.
(TIF)

Figure S6 AMPK and TSC2 are phosphorylated in RBS cells independent of p53. (A, B). By Western blot analysis, phosphorylation of AMPK and its substrate ACC was upregulated in human RBS cells, accompanied by increased TSC2 phosphorylation. (C). p53 knockdown did not affect AMPK or TSC2 phosphorylation in the RBS cells. (D and E). RBS cells produced an increase in reactive oxygen species (ROS) compared with WT or Corrected cells. P<0.001, ESCO2-Mut *vs* ESCO2-WT/Cor. For untransformed HSF or AFC, P<0.001, ESCO2-Mut *vs* ESCO2-WT.
(TIF)

Figure S7 The rescue of RBS zebrafish depends on ESCO2 mRNA, L-leucine and L-glutamine. (A, B). The specificity of the phenotype in ESCO2-morphant and mutant embryos was tested by injection of in vitro transcribed RNA encoding ESCO2 protein. Control injections were performed with in vitro transcribed control RNA. Scale bar = 200 µm. (C). Embryos (1–2 cells) were injected with ESCO2-5mis or ESCO2-Splice MO with control-mRNA or ESCO2-mRNA co-injection and treated with 200 nM rapamycin. After 2 d.p.f., the ESCO2-splice MO embryos looked more defective than those in which the ESCO2 mRNA was co-injected. (D). Embryos (1–2 cells) were injected with ESCO2-5mis or ESCO2-Splice MO (10 ng), and co-injected with a control mRNA or ESCO2 mRNA. After 5 d.p.f., the embryos were stained with Alcian blue to detect cartilage development. Scale bar = 200 µm. While the image is representative, about 15 embryos were analyzed per group. (E). Cranial development was quantified using the sum of the pq (palatoquadrate) cartilage and mc (Meckel's cartilage) divided by cranial length. The measurement was done on 3 embryos per group. P<0.001, ESCO2-MO with Control-mRNA co-injection *vs* ESCO2-5mis with Control-mRNA co-injection; P<0.001, ESCO2-MO with ESCO2-mRNA co-injection *vs* ESCO2-MO with Control-mRNA co-injection. (F). Embryos (1–2 cells) were injected with ESCO2-5mis or ESCO2-Splice MO (10 ng), and immediately separated into egg water with L-Glutamine (L-Glu) alone (4 mM), L-Leu alone (10 mM), or L-Leu (10 mM) plus L-Glu (4 mM) treatment. After 3 d.p.f., optimal rescue was observed with L-Leu plus L-Glu. Scale bar = 200 µm. (G). Embryos (1–2 cells) were injected with ESCO2-5mis or ESCO2-Splice MO (10 ng), and immediately separated into egg water with D-His (10 mM), D-Leu (10 mM), L-Thr (10 mM) or L-Leu (10 mM) treatment. All treatments included L-Glu (4 mM). After 3 d.p.f., only embryos treated with L-Leu showed partial improvement of development. Scale bar = 200 µm.
(TIF)

Figure S8 The effects of p53 knockdown on a zebrafish RBS model. (A). Embryos (1–2 cells) were co-injected with ESCO2-5mis or ESCO2-Splice MO (10 ng), and a control morpholino (Control-MO) from Gene Tools or p53 morpholino (p53-MO). After 2 d.p.f., the embryos were photographed. p53 knockdown showed some rescue of development in ESCO2 morphant embryos. Bar = 200 µm. (B). WT or ESCO2 mutant embryos were injected with Control-MO or p53-MO. After 2 d.p.f., the embryos were photographed. p53 knockdown showed some rescue of development in ESCO2 mutant embryos. (C). Embryos (1–2 cells) were co-injected with ESCO2-5mis or ESCO2-Splice MO (10 ng), and Control-MO or p53-MO. After 2 d.p.f. the embryos were harvested and analyzed by Western blot. p53-MO injection reduced p53 levels relative to the control MO in both the ESCO2-5mis and splice MO. Tubulin serves as a loading control. (D). Embryos were treated as in (C). Caspase 3/7 activity was measured as in Figure 7. p53 knockdown did not affect caspase activity in the ESCO2-splice MO. P<0.0001, ESCO2-Splice MO+Control MO *vs* Uninjected+Control MO or ESCO2-5mis+Control MO; P = 0.1307, ESCO2-Splice MO+p53 MO *vs* ESCO2-Splice MO+Control MO. (E and F). Embryos (1–2 cells) were co-injected with ESCO2-5mis or ESCO2-Splice MO (10 ng), and a control morpholino (Control-MO) from Gene Tools or p53 morpholino (p53-MO). After 5 d.p.f., the embryos were stained with alcian blue and the craniofacial length was quantified as in Figure 7. P<0.01, ESCO2-5mis+Control-MO *vs* ESCO2-MO+Control-MO; P = 0.336, ESCO2-MO+Control-MO *vs* ESCO2-MO+p53-MO.
(TIF)

Figure S9 p53 inhibition rescues high levels of phospho-histone H3 staining in ESCO2 morphants. (A). Embryos (1–2 cells) were co-injected with ESCO2-5mis or ESCO2-Splice MO (10 ng), and a control morpholino (Control-MO) from Gene Tools or p53 morpholino (p53-MO). At 24 h.p.f., the embryos were dechorionated and immunostained with anti-phospho-Histone H3 antibody to detect mitotic cells in G2/M stage. (B). The number of phospho-histone H3 positive cells was quantified for 5 embryos per group. P<0.0001, ESCO2-5mis+Control-MO *vs* ESCO2-MO+Control-MO; P<0.0001, ESCO2-MO+Control-MO *vs* ESCO2-MO+p53-MO.
(TIF)

Figure S10 Genotyping ESCO2-transgenic mutant and WT zebrafish embryos with PCR analysis. The ESCO2 mutant embryos have only the insert-bearing chromosome (only one 390 bp band), while the WT embryos have a non-transgenic chromosome that will give a 470 bp band.
(TIF)
