# Supplementary figures and images for: Stimulation of mTORC1 with L-leucine Rescues Defects Associated with Roberts Syndrome
Source: PLoS Genet. 2013 Oct 3;9(10):e1003857. doi: 10.1371/journal.pgen.1003857 (PMC3789817; doi:10.1371/journal.pgen.1003857)

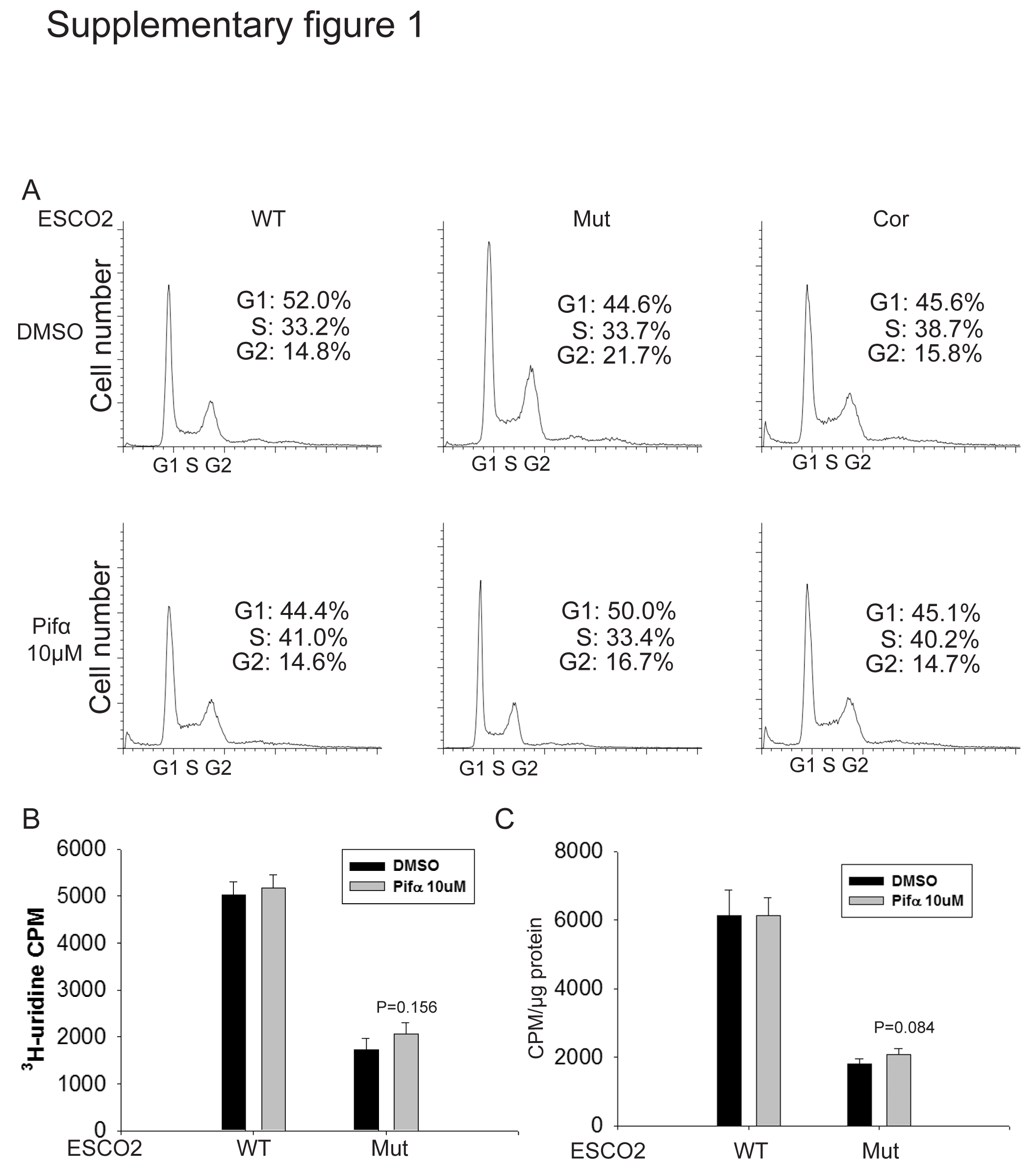

Supplement: Figure S1 — Inhibition of p53 did not rescue rRNA production or protein synthesis in human RBS cells. (A). The immortal WT, ESCO2-mutant and corrected cells were treated with p53 inhibitor Pifα (10 µM) or DMSO for 24 hrs, and FACScan analysis was performed with cell cycle measurements. (B). Untransformed WT and RBS fibroblasts were cultured in DMEM plus 10% FBS, in the presence of Pifα (10 µM) or DMSO for 24 hrs. 3H-uridine labeling experiments showed that rRNA production of RBS cells was not rescued by Pifα treatment. Each bar represents the average ± SEM of the ratio of the measurement of the indicated 3H-uridine incorporation into rRNA, as calculated for three independent samples. P = 0.156, ESCO2-Mut+Pifα vs ESCO2-Mut+DMSO. (C). Untransformed WT and RBS fibroblasts were cultured in DMEM plus 10% FBS, in the presence of Pifα (10 µM) or DMSO for 24 hrs. 35S-methionine labeling showed that protein synthesis in RBS cells was not rescued by Pifα treatment. Each bar represents the average ± SEM of the ratio of the measurement of the indicated 35S-methionine incorporation, as calculated for three independent samples. P = 0.084, ESCO2-Mut+Pifα vs ESCO2-Mut+DMSO. (TIF) [file pgen.1003857.s001.tif]

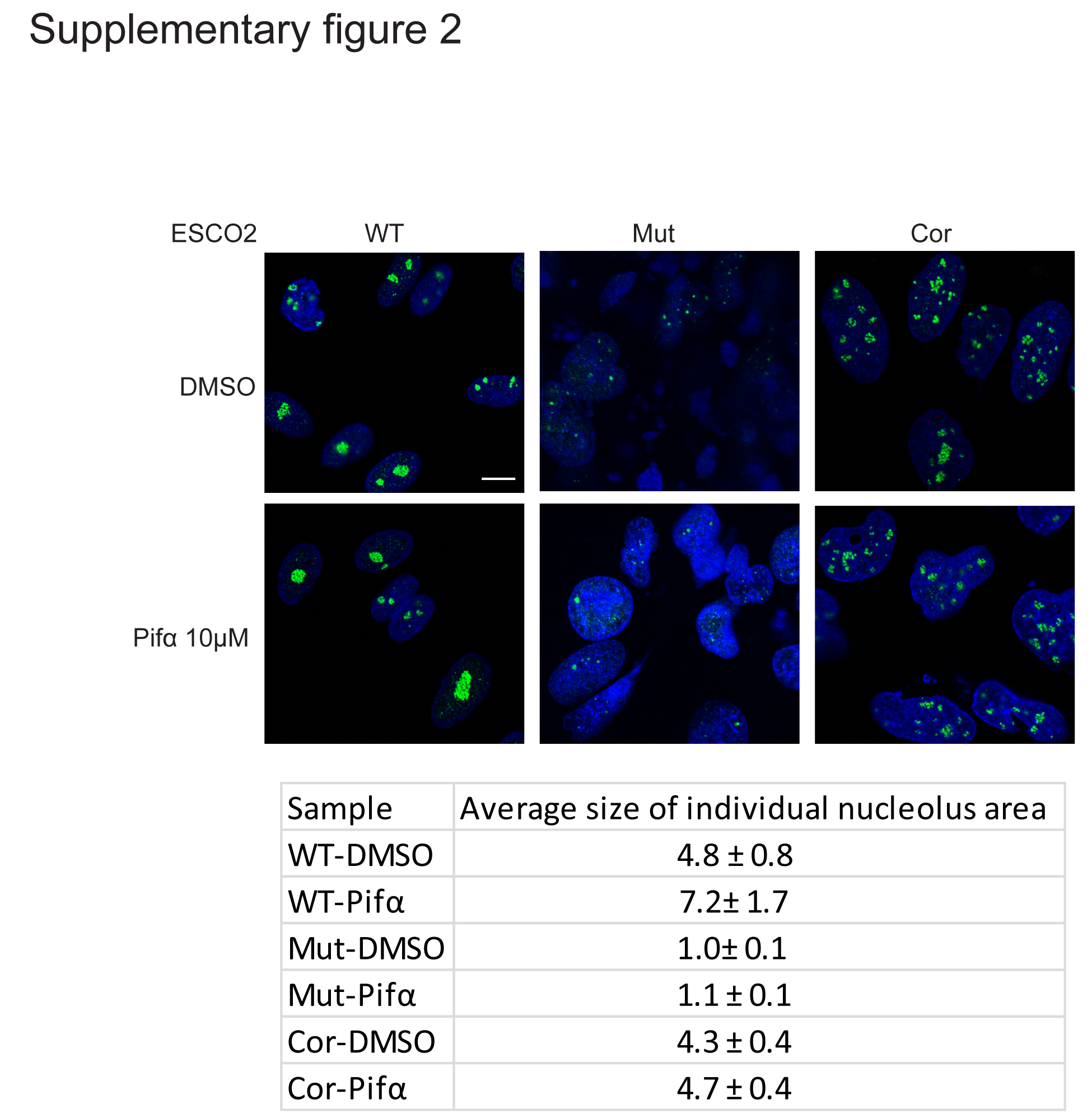

Supplement: Figure S2 — Nucleolar fragmentation was not rescued by inhibition of p53 in RBS cells. WT, RBS and corrected RBS cells were cultured in DMEM plus 10% FBS with p53 inhibitor Pifα (10 µM) or DMSO for 24 hrs. Cells were immunostained with anti-fibrillarin antibody, and imaged with confocal microscopy. DNA was stained with DAPI. The quantification of the nucleolar area was performed as in Figure 3. Bar = 10 µm. About 20 cells were quantified for each sample. (TIF) [file pgen.1003857.s002.tif]

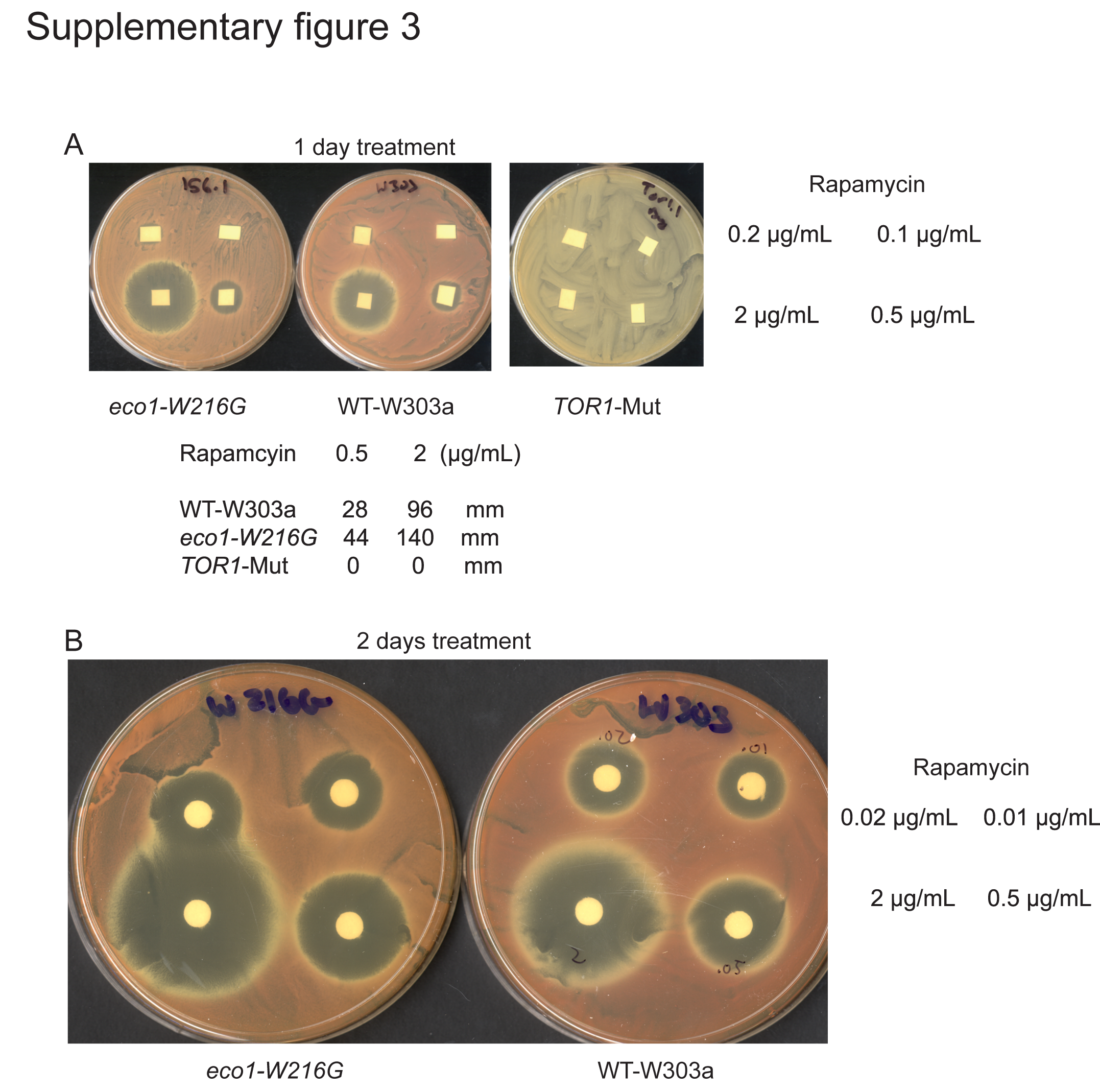

Supplement: Figure S3 — The eco1-W216G yeast mutant is hypersensitive to rapamycin. (A). Cultures of the indicated genotype were spread onto a plate and discs with the indicated amount of rapamycin were placed on the plate. The zone in which the yeast do not grow, or “halo,” showed that growth of the eco1-W216G mutant was hypersensitive to rapamycin. The diameter of each halo was measured at 1 day, and the growth of eco1-W216G mutant cells was reduced by about 50% compared with WT cells. The tor1 mutant was used as a control for a strain that is not sensitive to rapamycin. (B). The experiment in (A) was repeated and the halo was measured at 2 days. (TIF) [file pgen.1003857.s003.tif]

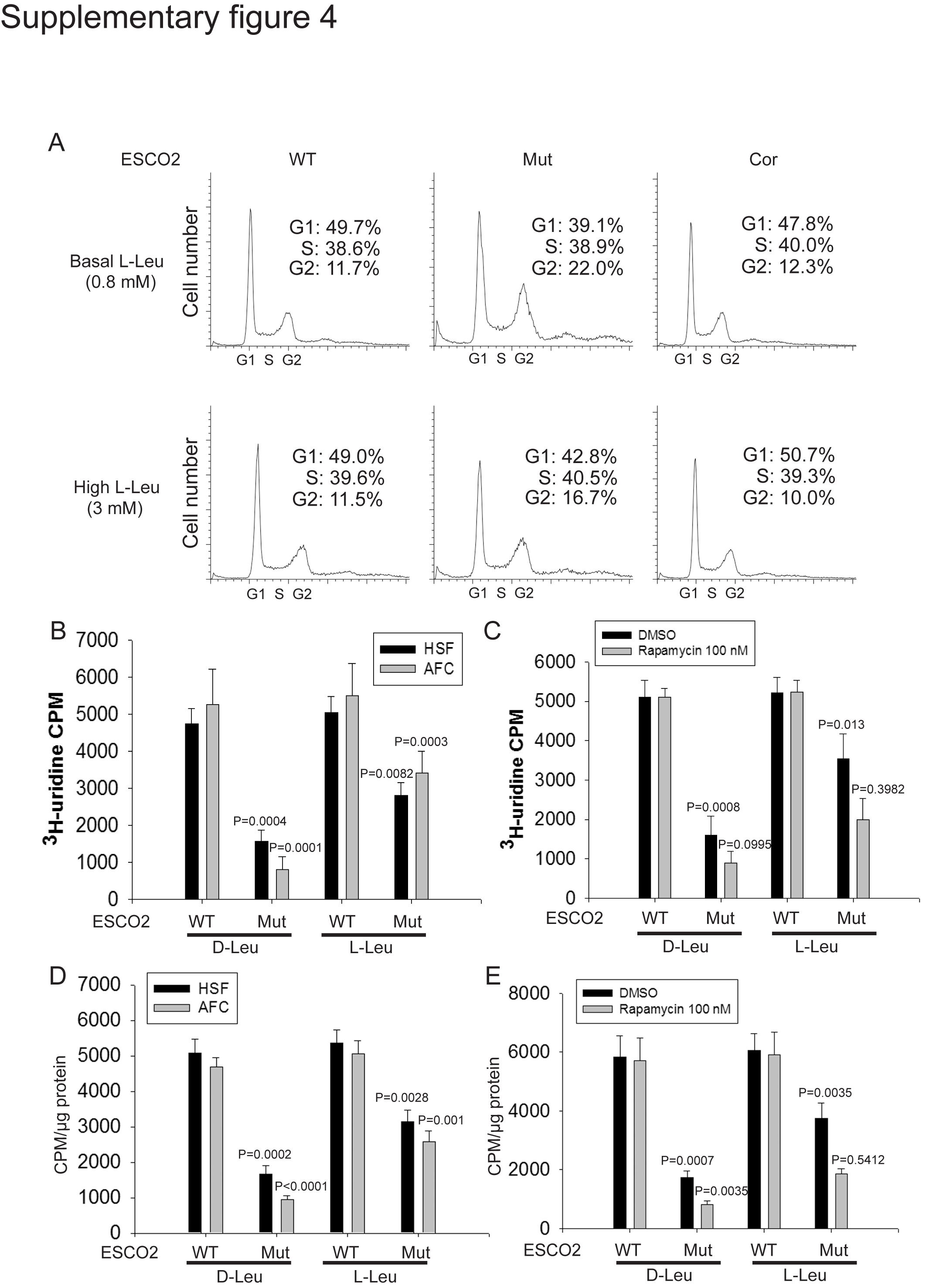

Supplement: Figure S4 — L-leucine rescues G2/M delay, rRNA production, and protein synthesis in RBS cells. (A). The immortal WT, ESCO2-mutant and corrected cells were cultured in the presence of basal levels of L-leucine (0.8 mM) or high levels of L-leucine (3 mM) for 24 hrs, and FACScan analysis with cell cycle measurement was performed. (B). WT and RBS cells were cultured in leucine-free DMEM plus 10% FBS, and supplemented with D-Leu or L-Leu (10 mM) for 24 hrs. 3H-uridine labeling showed that L-Leu partially rescued rRNA production in RBS cells. Each bar represents the average ± SEM of the ratio of the measurement of the indicated 3H-uridine incorporation into rRNA, as calculated for three independent samples. P = 0.0004, HSF ESCO2-Mut+D-Leu vs HSF ESCO2-WT+D-Leu; P = 0.0001, AFC ESCO2-Mut+D-Leu vs AFC ESCO2-WT+D-Leu; P = 0.0082, HSF ESCO2-Mut+L-Leu vs HSF ESCO2-Mut+D-Leu; P = 0.0003, AFC ESCO2-Mut+L-Leu vs AFC ESCO2-Mut+D-Leu. (C). WT and RBS cells were cultured as in (A) with or without 100 nM rapamycin. 3H-uridine labeling experiments showed that L-Leu partially rescued rRNA production of RBS cells via a mTORC1-dependent pathway. P = 0.0008, ESCO2-Mut+D-Leu with DMSO vs ESCO2-WT+D-Leu with DMSO; P = 0.0995, ESCO2-Mut+D-Leu with DMSO vs ESCO2-WT+D-Leu with rapamycin; P = 0.013, ESCO2-Mut+L-Leu with DMSO vs ESCO2-Mut+D-Leu with DMSO; P = 0.3982, ESCO2-Mut+L-Leu with rapamycin vs ESCO2-Mut+D-Leu with DMSO. (D). WT and RBS cells were cultured as in (A). 35S-methionine labeling showed that L-Leu partially rescued protein synthesis in RBS cells. Each bar represents the average ± SEM of the ratio of the measurement of the indicated 35S-methionine incorporation, as calculated for three independent samples. P = 0.0002, HSF ESCO2-Mut+D-Leu vs HSF ESCO2-WT+D-Leu; P<0.0001, AFC ESCO2-Mut+D-Leu vs AFC ESCO2-WT+D-Leu; P = 0.0028, HSF ESCO2-Mut+L-Leu vs HSF ESCO2-Mut+D-Leu; P = 0.001, AFC ESCO2-Mut+L-Leu vs AFC ESCO2-Mut+D-Leu. (E). WT and RBS cells were cultured as in (B). 35S-methionine labeling [file pgen.1003857.s004.tif]

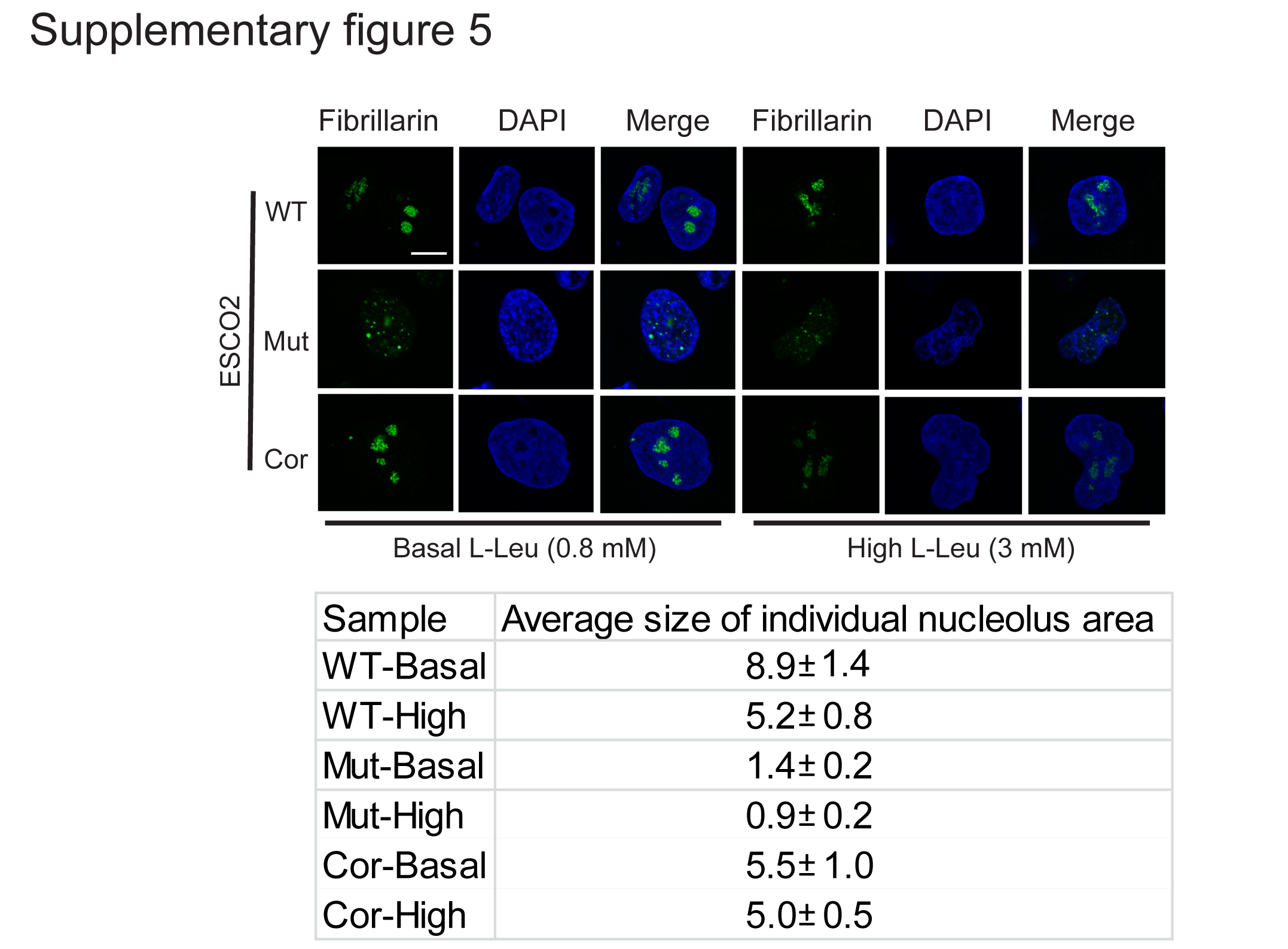

Supplement: Figure S5 — Nucleolar organization was not rescued by L-leucine treatment in immortalized RBS cells. WT, RBS and corrected RBS cells were cultured in DMEM plus 10% FBS with basal levels of L-Leu (0.8 mM in regular medium) or high levels of L-Leu (3 mM) for 2 days. Cells were immunostained with anti-fibrillarin antibody, and imaged with confocal microscopy. DNA was stained with DAPI. The quantification of the nucleolar area was performed as in Figure 3. Bar = 10 µm. About 20 cells were quantified for each sample. (TIF) [file pgen.1003857.s005.tif]

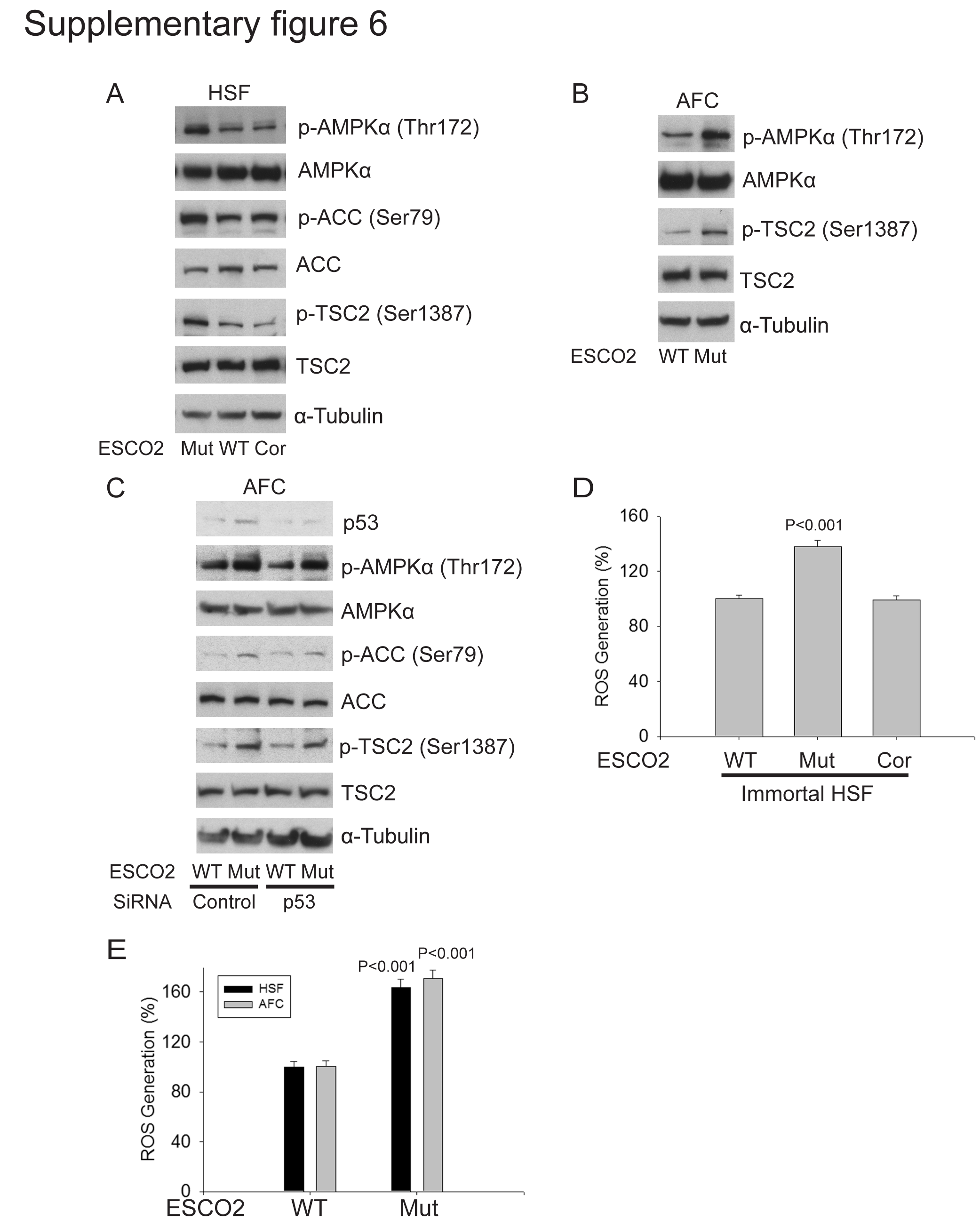

Supplement: Figure S6 — AMPK and TSC2 are phosphorylated in RBS cells independent of p53. (A, B). By Western blot analysis, phosphorylation of AMPK and its substrate ACC was upregulated in human RBS cells, accompanied by increased TSC2 phosphorylation. (C). p53 knockdown did not affect AMPK or TSC2 phosphorylation in the RBS cells. (D and E). RBS cells produced an increase in reactive oxygen species (ROS) compared with WT or Corrected cells. P<0.001, ESCO2-Mut vs ESCO2-WT/Cor. For untransformed HSF or AFC, P<0.001, ESCO2-Mut vs ESCO2-WT. (TIF) [file pgen.1003857.s006.tif]

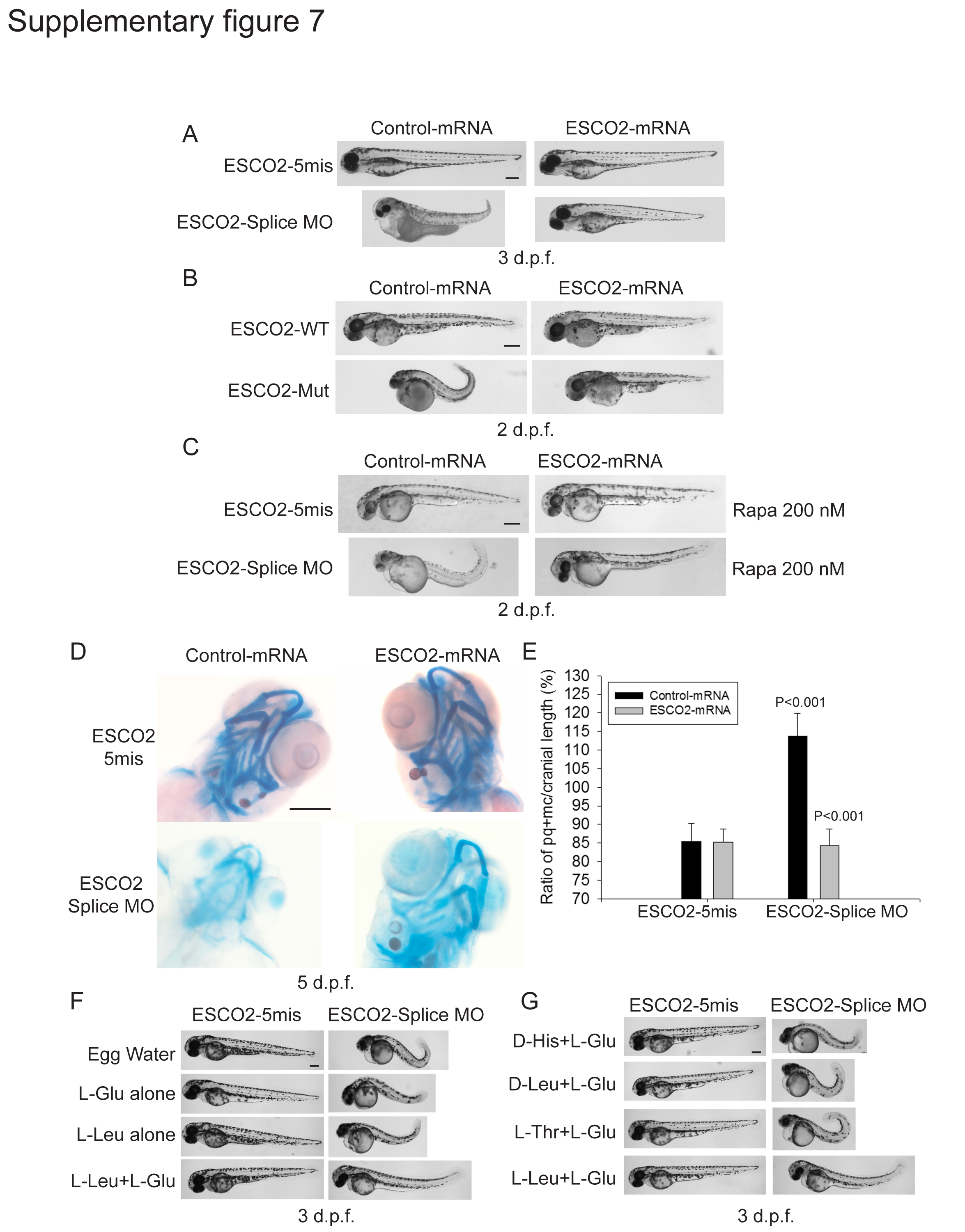

Supplement: Figure S7 — The rescue of RBS zebrafish depends on ESCO2 mRNA, L-leucine and L-glutamine. (A, B). The specificity of the phenotype in ESCO2-morphant and mutant embryos was tested by injection of in vitro transcribed RNA encoding ESCO2 protein. Control injections were performed with in vitro transcribed control RNA. Scale bar = 200 µm. (C). Embryos (1–2 cells) were injected with ESCO2-5mis or ESCO2-Splice MO with control-mRNA or ESCO2-mRNA co-injection and treated with 200 nM rapamycin. After 2 d.p.f., the ESCO2-splice MO embryos looked more defective than those in which the ESCO2 mRNA was co-injected. (D). Embryos (1–2 cells) were injected with ESCO2-5mis or ESCO2-Splice MO (10 ng), and co-injected with a control mRNA or ESCO2 mRNA. After 5 d.p.f., the embryos were stained with Alcian blue to detect cartilage development. Scale bar = 200 µm. While the image is representative, about 15 embryos were analyzed per group. (E). Cranial development was quantified using the sum of the pq (palatoquadrate) cartilage and mc (Meckel's cartilage) divided by cranial length. The measurement was done on 3 embryos per group. P<0.001, ESCO2-MO with Control-mRNA co-injection vs ESCO2-5mis with Control-mRNA co-injection; P<0.001, ESCO2-MO with ESCO2-mRNA co-injection vs ESCO2-MO with Control-mRNA co-injection. (F). Embryos (1–2 cells) were injected with ESCO2-5mis or ESCO2-Splice MO (10 ng), and immediately separated into egg water with L-Glutamine (L-Glu) alone (4 mM), L-Leu alone (10 mM), or L-Leu (10 mM) plus L-Glu (4 mM) treatment. After 3 d.p.f., optimal rescue was observed with L-Leu plus L-Glu. Scale bar = 200 µm. (G). Embryos (1–2 cells) were injected with ESCO2-5mis or ESCO2-Splice MO (10 ng), and immediately separated into egg water with D-His (10 mM), D-Leu (10 mM), L-Thr (10 mM) or L-Leu (10 mM) treatment. All treatments included L-Glu (4 mM). After 3 d.p.f., only embryos treated with L-Leu showed partial improvement of development. Scale bar = 200 µm. (TIF) [file pgen.1003857.s007.tif]

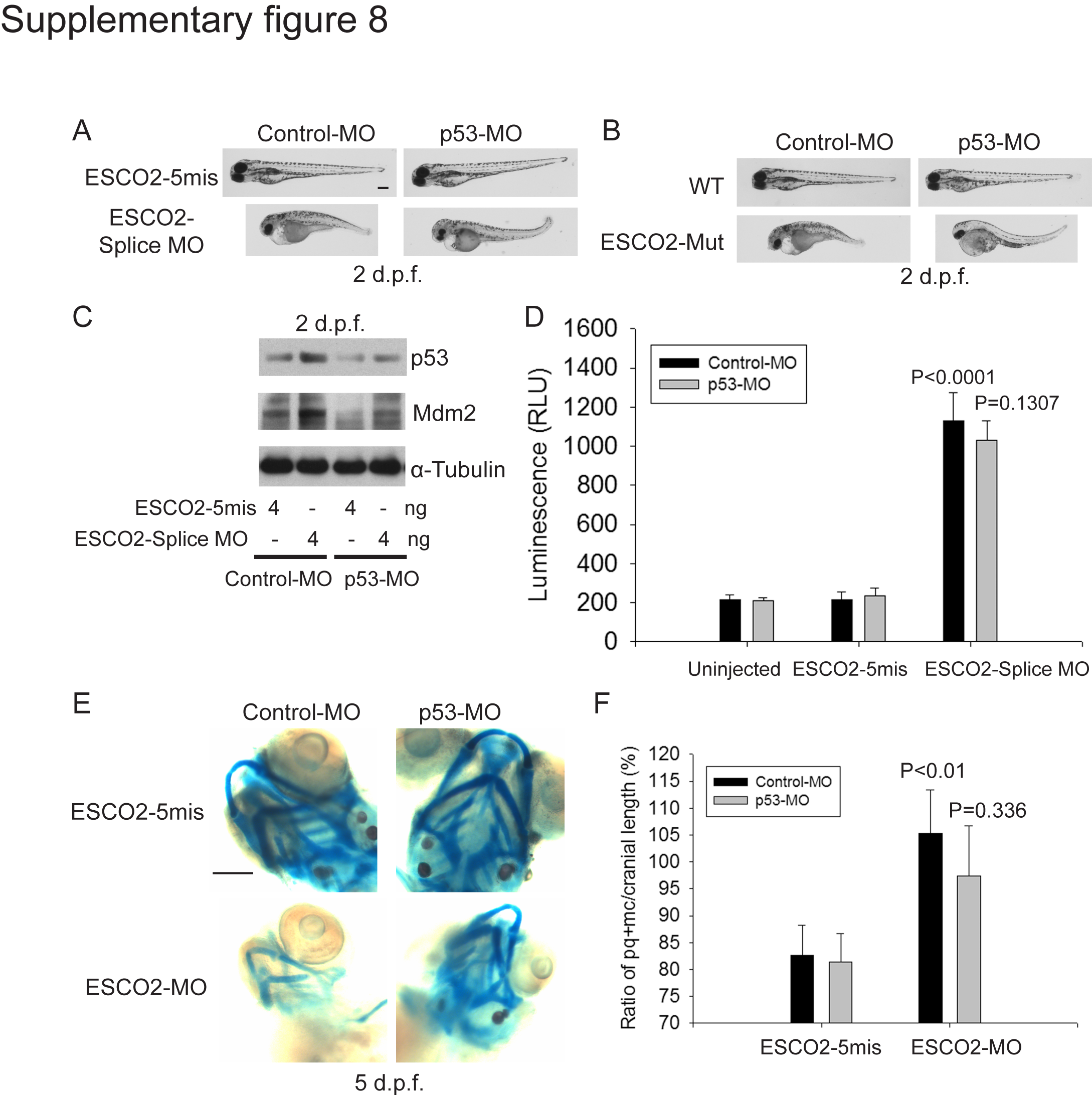

Supplement: Figure S8 — The effects of p53 knockdown on a zebrafish RBS model. (A). Embryos (1–2 cells) were co-injected with ESCO2-5mis or ESCO2-Splice MO (10 ng), and a control morpholino (Control-MO) from Gene Tools or p53 morpholino (p53-MO). After 2 d.p.f., the embryos were photographed. p53 knockdown showed some rescue of development in ESCO2 morphant embryos. Bar = 200 µm. (B). WT or ESCO2 mutant embryos were injected with Control-MO or p53-MO. After 2 d.p.f., the embryos were photographed. p53 knockdown showed some rescue of development in ESCO2 mutant embryos. (C). Embryos (1–2 cells) were co-injected with ESCO2-5mis or ESCO2-Splice MO (10 ng), and Control-MO or p53-MO. After 2 d.p.f. the embryos were harvested and analyzed by Western blot. p53-MO injection reduced p53 levels relative to the control MO in both the ESCO2-5mis and splice MO. Tubulin serves as a loading control. (D). Embryos were treated as in (C). Caspase 3/7 activity was measured as in Figure 7. p53 knockdown did not affect caspase activity in the ESCO2-splice MO. P<0.0001, ESCO2-Splice MO+Control MO vs Uninjected+Control MO or ESCO2-5mis+Control MO; P = 0.1307, ESCO2-Splice MO+p53 MO vs ESCO2-Splice MO+Control MO. (E and F). Embryos (1–2 cells) were co-injected with ESCO2-5mis or ESCO2-Splice MO (10 ng), and a control morpholino (Control-MO) from Gene Tools or p53 morpholino (p53-MO). After 5 d.p.f., the embryos were stained with alcian blue and the craniofacial length was quantified as in Figure 7. P<0.01, ESCO2-5mis+Control-MO vs ESCO2-MO+Control-MO; P = 0.336, ESCO2-MO+Control-MO vs ESCO2-MO+p53-MO. (TIF) [file pgen.1003857.s008.tif]

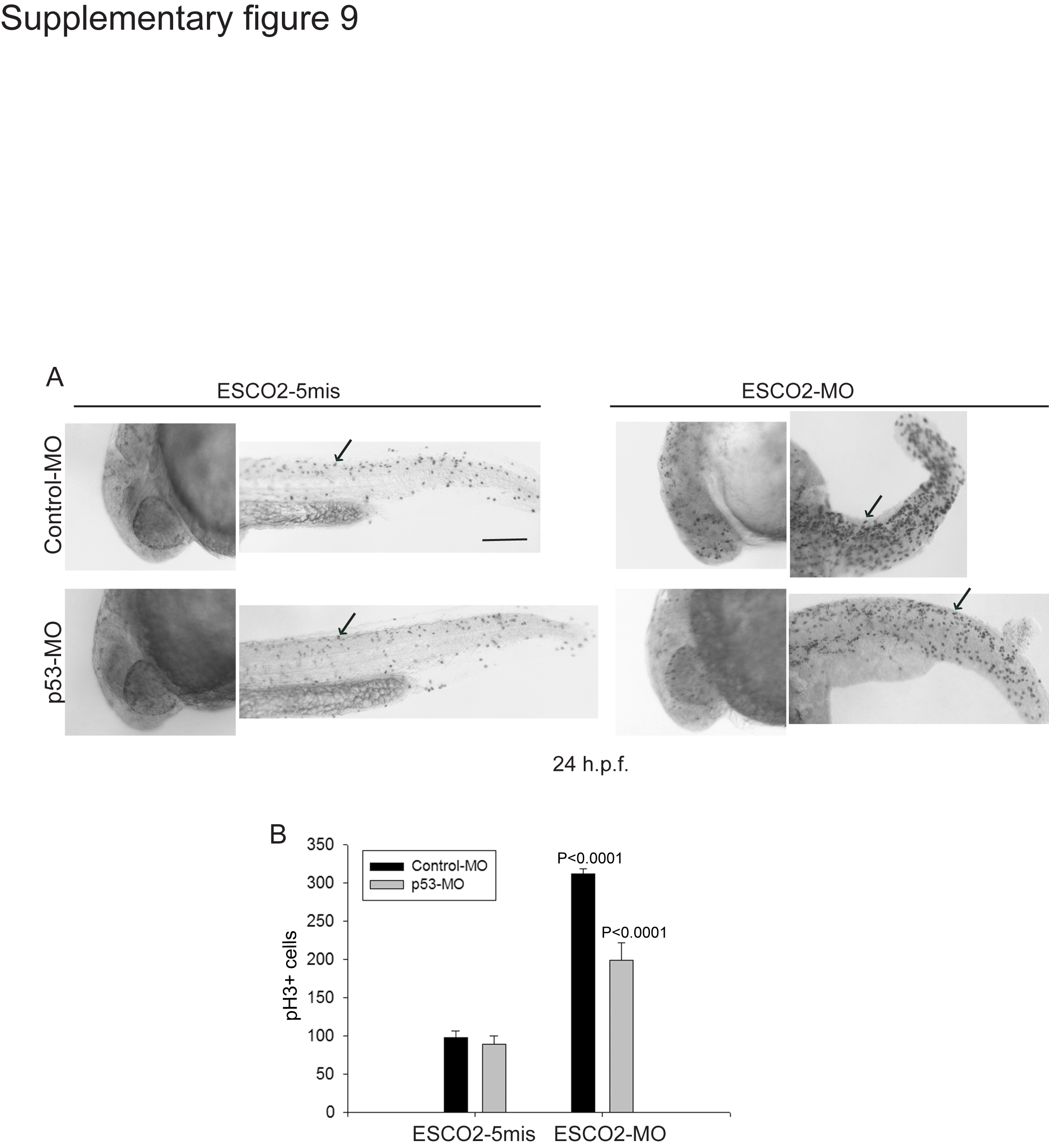

Supplement: Figure S9 — p53 inhibition rescues high levels of phospho-histone H3 staining in ESCO2 morphants. (A). Embryos (1–2 cells) were co-injected with ESCO2-5mis or ESCO2-Splice MO (10 ng), and a control morpholino (Control-MO) from Gene Tools or p53 morpholino (p53-MO). At 24 h.p.f., the embryos were dechorionated and immunostained with anti-phospho-Histone H3 antibody to detect mitotic cells in G2/M stage. (B). The number of phospho-histone H3 positive cells was quantified for 5 embryos per group. P<0.0001, ESCO2-5mis+Control-MO vs ESCO2-MO+Control-MO; P<0.0001, ESCO2-MO+Control-MO vs ESCO2-MO+p53-MO. (TIF) [file pgen.1003857.s009.tif]

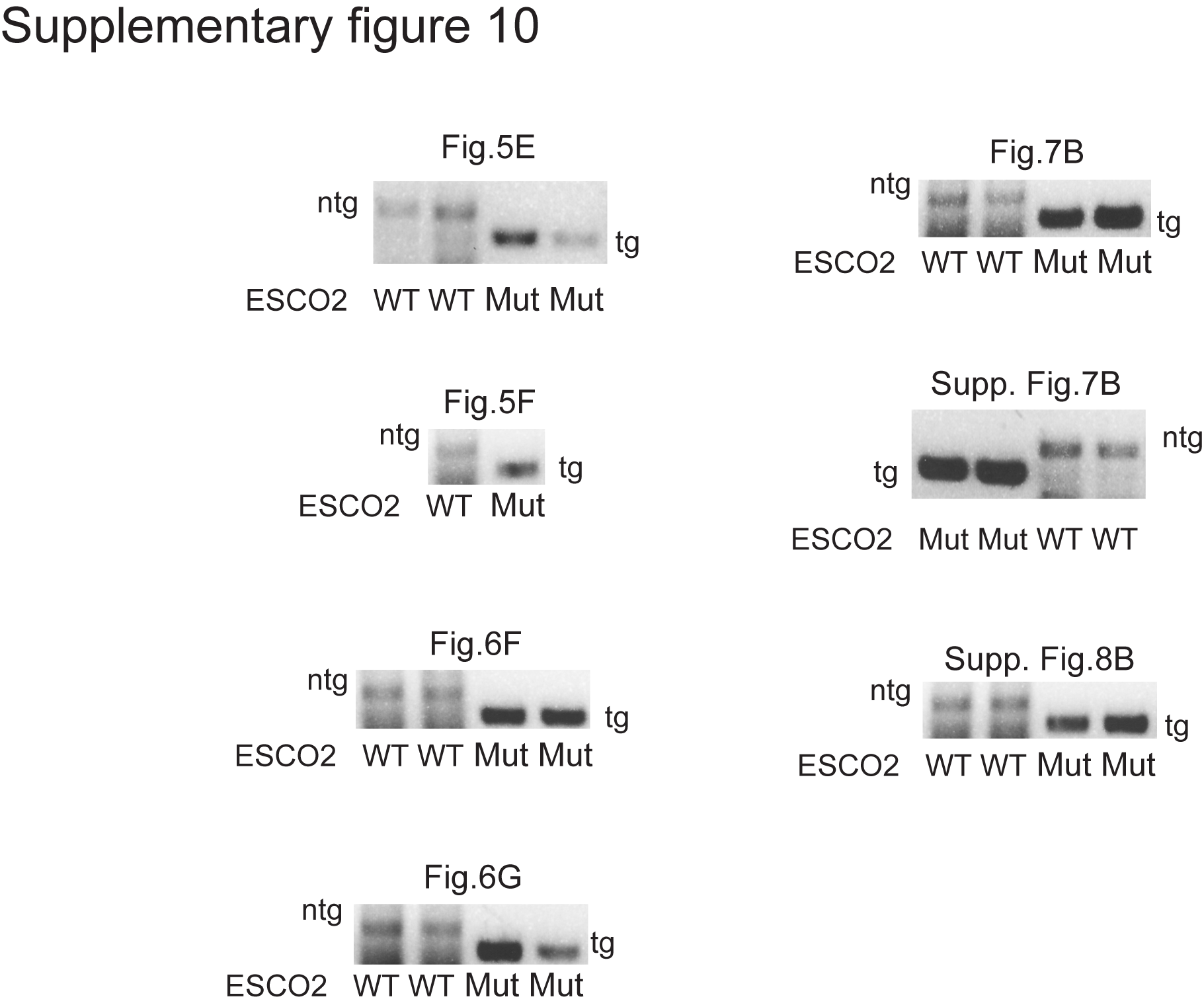

Supplement: Figure S10 — Genotyping ESCO2-transgenic mutant and WT zebrafish embryos with PCR analysis. The ESCO2 mutant embryos have only the insert-bearing chromosome (only one 390 bp band), while the WT embryos have a non-transgenic chromosome that will give a 470 bp band. (TIF) [file pgen.1003857.s010.tif]
